# Genetic Determinants of Interindividual Differences in Provitamin A Carotenoid Concentrations in Human Adipose Tissue

**DOI:** 10.1002/mnfr.70557

**Published:** 2026-07-27

**Authors:** Patrick Borel, Mark Pretzel Zumaraga, Charles Desmarchelier

**Affiliations:** ^1^ C2VN Aix Marseille Univ INRAE INSERM Marseille France; ^2^ Department of Science and Technology – Food and Nutrition Research Institute Taguig City Philippines; ^3^ Institut Universitaire de France (IUF) Paris France

**Keywords:** lipid, nutrigenetics, single nucleotide polymorphisms, α‐carotene, β‐carotene, β‐cryptoxanthin

## Abstract

Adipose tissue is a major storage site for provitamin A carotenoids (proVA CAR)—mainly β‐carotene (BCAR), α‐carotene (ACAR), and β‐cryptoxanthin (BCRY). However, the determinants of their concentrations in this tissue remain poorly understood. This study aimed to identify genetic variants associated with adipose tissue proVA CAR concentrations. Periumbilical adipose tissue samples were collected on six occasions in 43 healthy adult males and proVA CAR concentrations were quantified by HPLC. Participants were genotyped for 2,398 SNPs in 44 candidate genes involved in CAR and lipid metabolism, and genetic associations were assessed using partial least squares (PLS) regression. Adipose tissue proVA CAR concentrations ranked as BCAR > ACAR > BCRY, with CV ranging from 54 to 66%. PLS regression models integrating fasting plasma proVA CAR concentrations and SNPs explained 69, 59, and 67% of the variability in BCAR, ACAR, and BCRY concentrations, respectively. In a multivariate PLS regression model, the main determinants of proVA CAR concentrations were fasting plasma BCAR and ACAR concentrations and four SNPs—rs4694627 (*CXCL8*), rs7558381 (*IRS1*), rs709157 and rs1152004 (*PPARG*). Interindividual variability in adipose tissue proVA CAR concentrations is partly explained by genetic variation in genes involved in CAR and lipid metabolism.

**Clinical Trial Registry**: ClinicalTrials.gov registration number NCT02100774.

## Graphical abstract

Periumbilical adipose tissue provitamin A carotenoids (proVA CAR) were quantified in 43 healthy adult males to investigate genetic factors associated with their tissue concentrations. β‐Carotene was the most abundant adipose tissue proVA CAR, followed by α‐carotene and β‐cryptoxanthin, with marked interindividual variability. Multivariate modeling identified that fasting plasma β‐carotene and α‐carotene concentrations together with genetic variants in *PPARG*, *IRS1*, and *CXCL8* as the main determinants of adipose tissue proVA CAR concentrations. These findings indicate that genetic variation contributes to interindividual variability in adipose tissue proVA CAR storage.

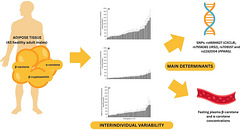

**Abbreviations**
ACAR: α‐carotene
BCAR: β‐carotene
BCO1: β‐carotene oxygenase 1
BCRY: beta‐cryptoxanthin
CXCL8: chemokine (C–X–C motif) ligand 8
IRS1: insulin receptor substrate 1
ISX: intestine specific homeobox
PLS: partial least squares
PPARG: peroxisome proliferator activated receptor gamma
ProVA CAR: provitamin A carotenoid
SCARB1: scavenger receptor class B member 1

## 1 Introduction

**FIGURE 1:**
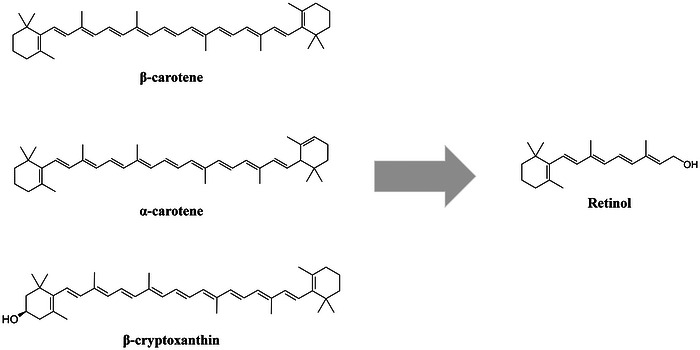
Structure of major provitamin A carotenoids. Carotenoids that possess at least one unsubstituted β‐ionone ring serve as the precursors for the synthesis of all‐*trans*‐retinol (commonly known as vitamin A). All‐*trans*‐β‐carotene is symmetrically cleaved by β‐carotene oxygenase 1 (BCO1) to yield two molecules of all‐*trans*‐retinal. All‐*trans*‐retinal is then reduced to all‐*trans*‐retinol by retinaldehyde reductases. All molecules are shown in their *trans* configuration.

Carotenoids (CAR) are a group of lipophilic pigments produced by most photosynthetic organisms, including plants, algae, and some bacteria and fungi. Humans cannot synthesize them and rely entirely on dietary intake to obtain them, mostly through the consumption of vegetables and fruits [1]. Of the several hundred naturally occurring CAR, around 50–60 have provitamin A (proVA) activity and are thus referred to as proVA CAR [2]. This activity is due to the presence of at least one unsubstituted β‐ionone ring, which enables central enzymatic cleavage by β‐carotene oxygenase 1 (BCO1) to yield retinol [3]. Only a few proVA CAR are found in significant amounts in the human diet [2], with β‐carotene (BCAR), α‐carotene (ACAR), and β‐cryptoxanthin (BCRY) being the main ones (Figure 1). These 3 proVA CAR are typically the only ones listed in food composition tables, with BCAR contributing 71%–75% of total proVA CAR intake, ACAR 13%–18%, and BCRY 6%–15% in high‐income countries [4, 5]. ProVA CAR make a significant contribution to total VA intake, estimated at 35% in high‐income countries [5]. This contribution is substantially higher in low‐income regions—for example, 70% in Africa and 75% in Asia [6]—primarily because of limited access to animal products. Similarly, in high‐income regions, individuals who voluntarily reduce or exclude products of animal origin (e.g., flexitarians, vegetarians, or vegans) may also rely more heavily on proVA CAR to meet their VA needs [7]. Beyond serving as a source of VA, proVA CAR could also exert other biological effects, although most studies focused on BCAR. For instance, higher dietary intake or circulating concentrations of BCAR have been associated with reduced risk of several chronic diseases, including type 2 diabetes, cardiovascular diseases (including stroke), and certain cancers [8, 9], and lower all‐cause mortality [10]. Additionally, studies in *bco1^−/−^* mice have demonstrated a protective effect of BCAR on atherosclerosis [11, 12].

In the human body, adipose tissue is considered, along with the liver, one of the main storage sites of CAR [13], where they are mostly stored in the lipid droplet of adipocytes [14]. Although specific data are lacking for proVA CAR, Moran et al. reported, based on 18 publications, that for another CAR, namely lycopene, adipose tissue accounts for 60%–72% of the total body pool [15]. Chung et al. quantified major CAR in three adipose depots (abdominal, gluteal, and femoral) of 25 U.S. adults. Overall, proVA CAR concentrations were highest in the abdomen, intermediate in the buttocks, and lowest in the thigh. The authors found a consistent ranking: lycopene > BCAR > BCRY > lutein > ACAR > zeaxanthin [16]. This predominance of lycopene likely reflects the high consumption of tomatoes in the U.S.  [1, 17]. In contrast, in 14 Australian adults, BCAR was the most abundant CAR in abdominal adipose tissue (BCAR > ζ‐carotene > phytofluene > ACAR > lycopene > phytoene > lutein), highlighting the quantitative importance of proVA CAR in adipose tissue [18]. Although long hypothesized [19], proVA CAR conversion to VA by BCO1 in the adipose tissue was only recently demonstrated [20]. However, the extent to which this pathway contributes to local or systemic VA status remains unknown. In adipose tissue, proVA CAR (most research concerns BCAR) have been shown to inhibit adipogenesis [20, 21], notably through the transcriptional effects of some of their metabolites, likely retinoic acid [20, 22]. Moreover, in this tissue, proVA CAR and/or some of their metabolites are also involved in regulating oxidative stress, as well as influencing the production of regulatory signals and inflammatory mediators [23, 24].

Despite their physiological relevance, the factors influencing proVA CAR concentrations in adipose tissue remain poorly understood [1]. Although adipose tissue CAR concentrations have been reported to be similar between males and females [1], some have shown higher concentrations in females [25]. They have been shown to be only partly correlated to their dietary intakes [16, 26] or circulating concentrations [16, 18, 26]. They have also been reported to be inversely correlated with BMI [18, 27]. Previous work from our group [P.B.] in rats has indicated that adipose tissue accumulation of proVA CAR is not simply determined by their physicochemical characteristics [28], implying the involvement of protein‐mediated transport mechanisms. Consistent with this, in vitro and ex vivo studies have shown that the membrane protein CD36 molecule (CD36), which is widely expressed across cell types, facilitates the uptake of CAR into adipocytes [29]. Based on these observations, we hypothesized that genetic variations in genes encoding proteins involved in CAR transport and metabolism could contribute to the observed interindividual differences in adipose tissue proVA CAR concentrations.

Therefore, the objectives of this study were twofold: (1) to characterize interindividual variability in proVA CAR concentrations within subcutaneous white adipose tissue in a sample of 43 healthy adult males, and (2) to identify single nucleotide polymorphisms (SNPs) associated with adipose tissue proVA CAR concentrations using a candidate gene approach.

## 2 Methods

### 2.1 Participants

To maximize the ability to detect genetic determinants of adipose tissue proVA CAR concentrations, the study was restricted to healthy males in order to limit biological variability unrelated to genetics, including sex‐related differences in adiposity, lipid metabolism, and hormonal regulation. Forty‐three healthy, nonoverweight, nonobese, and nonsmoking adult males participated in this study. The exclusion criteria included a history of chronic disease, hyperlipidemia, or hyperglycemia, as well as the use of any medication or dietary supplement that might affect lipid metabolism (e.g., tetrahydrolipstatin, ezetimibe, cholestyramine, fibrates, and statins) within 1 month prior to or during the study. Because of the relatively large volume of blood drawn during the study, participants were required to have a blood hemoglobin concentration >130 g L^−1^ as an additional inclusion criterion. Their baseline characteristics are shown in Table 1. The study was approved by the regional committee on human experimentation (N°2008‐A01354‐51, Comité de Protection des Personnes Sud Méditerranée I, France). Procedures followed were in accordance with the Declaration of Helsinki of 1975 as revised in 1983. Informed written consent was obtained from each participant.

**TABLE 1:** Baseline characteristics of the study participants (*n* = 43).

Characteristic	Mean (SEM)
Age, y	31.9 (1.9)
BMI, kg m^−2^	23.0 (0.3)
Lipid profile	
Total cholesterol, g L^−1^ ^a^	1.7 (0.1)
Triglycerides, g L^−1^ ^a^	0.8 (0.1)
LDL‐C, g L^−1^ ^a^	1.1 (0.1)
HDL‐C, g L^−1^ ^a^	0.5 (0.0)
Glucose, mmol L^−1^ ^a^	4.7 (0.1)
Hemoglobin, g dL^−1^ ^a^	15.1 (0.1)
BCAR, µmol L^−1^ ^a^	0.300 (0.028)
BCRY, µmol L^−1^ ^a^	0.178 (0.020)
ACAR, µmol L^−1^ ^a^	0.111 (0.013)

Abbreviations: ACAR, α‐carotene; BCAR, β‐carotene; BCRY, β‐cryptoxanthin

^a^ Analytes were quantified in fasting plasma samples.

### 2.2 Study Design

This study was a secondary analysis of a randomized cross‐over clinical trial. Since no study investigating the association between genetic variations and adipose tissue proVA CAR concentrations has been published, it was not possible to carry out a power calculation. Therefore, this study should be considered exploratory. The original aim of the trial was to identify combinations of SNPs associated with the interindividual variability of lipophilic micronutrient bioavailability, namely α‐tocopherol [30], cholecalciferol [31], β‐carotene [32], lycopene [33], and lutein [34]. To this end, participants were provided with five different test meals, containing or not the aforementioned micronutrients, and their postprandial chylomicron concentrations were measured. Moreover, adipose tissue samples were collected during the visits associated with the consumption of three of the five test meals: the control, the vitamin E, and the tomato puree meal. This selective approach was chosen to minimize participant dropout due to the relatively invasive nature of the procedure (*see* 2.3). The composition of the three test meals provided to the participants was presented elsewhere [35] and the only difference compared to the control meal was that the vitamin E meal was provided with a capsule containing *RRR*‐α‐tocopheryl acetate (equivalent to 67 mg α‐tocopherol), while the tomato puree meal had 100 g of tomato puree added. The first two meals were expected to have negligible amounts of proVA CAR while the tomato puree meal provided approximately 400 µg BCAR. The three test meals were taken in a random order and were separated by a washout period of at least 3 weeks. Two days before each test meal, participants were instructed to restrict their intake of foods high in CAR (they were provided with an exclusion list). Participants were instructed to eat dinner between 7 and 8 p.m. the day before each test meal, abstaining from all alcoholic beverages. In addition, they were instructed to limit their intake to water only after supper and until they arrived at the Center for Clinical Investigation (la Conception Hospital, Marseille, France). On the day of the clinical experiment, participants were instructed to eat the test meal at a consistent rate, i.e. half of the meal within the first 10 min and the second half within the next 10 min. After each test meal, participants were asked to refrain from consuming any other food for the next 8 h, except for the water provided with the meal.

### 2.3 Adipose Tissue Biopsies

Adipose tissue sampling has been described in detail elsewhere [35]. Briefly, subcutaneous adipose tissue samples from the periumbilical region were collected by a trained physician at two time points: in the fasting state and 8 h after the intake of the three test meals.

### 2.4 Fasting and Postprandial Biochemical Measurements

At baseline, fasting plasma concentrations of total cholesterol, HDL‐cholesterol, LDL‐cholesterol, triglyceride, glucose, and hemoglobin were measured using standard methods as previously described [36]: all analyte concentrations were measured on a Modular PP instrument (Roche Diagnostics, Meylan, France), except for hemoglobin, which was measured on an ADVIA 120 instrument (Siemens Healthcare Diagnostics, Saint‐Denis, France), at La Conception Hospital (Biochemistry Laboratory, Marseille). All analyses were performed following manufacturers’ instructions. Fasting plasma concentrations at baseline of proVA CAR (i.e. BCAR, ACAR, BCRY) were measured by HPLC, as described below.

Postprandial plasma chylomicron BCAR concentrations (from 1 to 8 h) following consumption of the tomato puree meal were measured as previously described [32].

### 2.5 ProVA CAR Extraction

Approximately 50 mg of adipose tissue (fresh weight) was crushed in 300 µL of phosphate‐buffered saline using an MM301 ball mill with two 3‐mm diameter stainless steel balls (Retsch, Eragny‐sur‐Oise, France). A 50 µL aliquot thereof was used for protein quantification (BiCinchoninic acid Assay kit, Pierce, Montluçon, France), after dilution to 1/5 in phosphate‐buffered saline buffer. The remaining 250 µL underwent lipid extraction using 2 mL trichloromethane/methanol (1/1, v/v) and 0.9 mL phosphate‐buffered saline. All extractions were conducted at room temperature under yellow light to minimize light‐induced damage to the proVA CAR. The dried extracts were incubated 1 h 30 min at 37 °C with 100 µL of an ethanolic pyrogallol solution (12%, w/v), to prevent oxidative damage to the studied compounds, and 1 mL of an ethanolic potassium hydroxide solution (5.5%, w/v). After cooling to room temperature, an internal standard (echinenone) was added. The mixture underwent two extractions with 3 mL of hexane. The resulting extract was evaporated under nitrogen and solubilized in 100 µL methanol/dichloromethane (65/35, v/v) for subsequent HPLC analysis.

### 2.6 HPLC Analysis

A volume of 90 µL was used for HPLC analysis. The separation was performed using a 10.0 × 4.0 mm Modulo‐Cart QS guard column with a 2‐µm particle size (Interchim), followed by a 250 × 4.6 mm YMC C30 analytical column with a 5‐µm particle size (Interchim), maintained at a constant temperature of 35 °C. The mobile phase consisted of HPLC‐grade methanol (component A), methyl *tert*‐butyl ether (component B), and water (component C) (Carlo Erba–SDS). A linear gradient was applied, starting with 96% A, 2% B, and 2% C at time 0, transitioning to 18% A, 80% B, and 2% C by 27 min, at a flow rate of 1 mL min^−1^. The HPLC system consisted of a pump (Waters 2690) associated with a photodiode‐array detector (Waters 2996) (Waters). Detection of proVA CAR was based on UV spectra and retention times matching those of authentic standards (kindly provided by Dr. Adrian Wyss, DSM Nutritional Products Ltd.), with quantification performed at 450 nm. Peak integration and quantification were carried out using Chromeleon CDS software (version 6.80, Dionex), using external calibration curves normalized to the internal standard.

### 2.7 DNA Extraction and Genotyping

Twenty‐five µg of DNA was extracted from saliva samples using the Oragene extraction kit (DNA Genotek, Ottawa, Ontario, Canada). DNA preparation and genotyping methods were performed as previously described [37]. The whole genome was genotyped using HumanOmniExpress BeadChips (Illumina, California, USA), which allow for the analysis of ≈7.33 × 10^5^ SNPs/DNA sample.

### 2.8 Candidate SNP Selection and SNP Function Prediction

Following a literature review, genes whose encoded proteins have been previously shown to influence proVA CAR concentration in adipose tissue, either directly, by being involved in their absorption by adipocytes or in their metabolism within these cells, or indirectly, by influencing blood proVA CAR concentration or by being involved in lipid metabolism within adipose tissue, were selected. Candidate genes therefore included genes involved in CAR absorption and metabolism, lipid metabolism in adipose tissue, circulating CAR concentrations identified by genome‐wide association studies, as well as genes whose SNPs had previously been associated with postprandial chylomicron CAR or triglyceride responses in the same cohort. This process resulted in the selection of 44 candidate genes (Table S1), corresponding to 4,616 SNPs on the DNA chips. SNPs that had a genotype call rate <95%, that presented a significant departure from Hardy–Weinberg equilibrium (*p* < 0.05; chi‐squared test), that were in high linkage disequilibrium (LD; *R* ^2^ > 0.80, according to the LD TAG SNP Selection tool from the SNPinfo Web Server, HapMap CEU population, accessible at https://snpinfo.niehs.nih.gov), or that had fewer than 5 observations per genotypic group were excluded from the analysis. SNPs were tested under both additive (793 SNPs) and dominant (2398 SNPs) models. Note that the same set of SNPs was tested for association with adipose tissue concentrations of BCAR, BCRY, and ACAR. The SNP selection flowchart is summarized in Figure S1.

In a secondary step, LDlink (https://ldlink.nih.gov/; CEU population from the 1000 Genomes Project) was queried to identify variants in linkage disequilibrium with SNPs significantly associated with adipose tissue proVA CAR concentrations, in order to determine whether these variants or their proxies had been previously reported in the literature and to facilitate biological interpretation of the observed associations.

PolyPhen‐2, integrated within the Ensembl Variant Effect Predictor (https://www.ensembl.org/Tools/VEP), was used to assess the potential impact of exonic variants on protein structure or function [38]. The PolyPhen‐2 score ranges from 0 to 1, with higher scores indicating a greater likelihood of functional impact. The potential functional effects of noncoding variants (intronic or intergenic) were predicted using RegulomeDB (v2.0; https://regulomedb.org). The RegulomeDB probability score ranges from 0 to 1, where a value of 1 indicates that the variant is more likely to be regulatory [39]. For interpretative purposes, scores were categorized as high (>0.7), moderate (0.4–0.7), or low (<0.4) regulatory probability.

### 2.9 Statistics

Data were expressed as mean ± SEM. Adipose tissue BCAR, BCRY, and ACAR concentrations, measured in the fasting state and 8 h after consumption of the three test meals, were first analyzed using linear mixed models. The models included meal (control, vitamin E, and tomato puree) and time (fasting and 8 h postprandial) as fixed within‐subject factors, and participant as the random factor. To account for repeated measures, five covariance structures (autoregressive order one, diagonal, scaled identity, unstructured, and compound symmetry) were tested, and the best‐fitting model was selected according to Akaike's Information Criterion (AIC). Model diagnostics were assessed using residual scatterplots (homoscedasticity) and QQ plots (normality).

Since neither meal nor time significantly influenced adipose tissue BCAR, BCRY, or ACAR concentrations, data within each participant were treated as technical replicates. Outliers among replicates were removed following identification using two‐tailed Grubbs’ tests (https://www.graphpad.com/quickcalcs/Grubbs1.cfm). Arithmetic means were then calculated for each participant, hereafter referred to as adipose tissue BCAR, ACAR, and BCRY concentrations. Relationships between these concentrations and anthropometric or plasma variables were assessed using Pearson's correlation coefficients. Coefficients of variation (CV) in plasma and adipose tissue proVA CAR concentrations were compared following Forkman [40]. All analyses were performed with SPSS 28 (SPSS Inc., Chicago, IL, USA), with the significance threshold set at *α* = 0.05.

To investigate genetic contributions, we applied partial least squares (PLS) regression to identify combinations of SNPs that best explained interindividual variability in adipose tissue proVA CAR concentrations. The same procedure was applied separately to each proVA CAR. A two‐step procedure was used, as previously described for SNP analyses [35, 36, 41, 42, 43]. First, for a given proVA CAR, univariate filtering was performed to select SNPs showing *p* < 0.05 (Wald test, PLINK v1.07: https://zzz.bwh.harvard.edu/plink/), in order to reduce the dimensionality of the predictor set and improve model stability prior to multivariate modeling. Second, a PLS regression model was built including these SNPs along with anthropometric and plasma variables that showed a nonzero correlation (Pearson's *r* ≠ 0) with the adipose tissue concentration of the given proVA CAR. These variables were entered as X variables, while the adipose tissue concentration of the given proVA CAR served as the Y variable. All variables were standardized (mean‐centered and scaled to unit variance) prior to modeling so that variables measured on different scales (e.g., genotypes, anthropometric data, and metabolite concentrations) contributed equally to the model and no variable dominated the latent components due to its numerical range.

Within PLS regressions, X variables were ranked according to their variable importance in projection (VIP) values, which estimate the contribution of each variable to the model. Several models were constructed using increasing VIP value thresholds [44]. The optimal number of latent components was determined automatically by SIMCA using its default cross‐validation procedure, in which additional components are retained only if they significantly improve the predictive ability of the model. All final models retained a single component. Cross‐validation was performed using the default 7‐fold procedure implemented in SIMCA [45, 46, 47]. The model that maximized the explained variance (adjusted *R* ^2^) and was statistically significant according to cross‐validation ANOVA [48] was selected.

The final model was further evaluated by internal validation procedures: leave‐*k*‐out cross‐validation [49], regression coefficient stability testing [37], and response permutation testing (*see* additional validations in the Supporting Information). For permutation testing, the explained and cross‐validated variance of the original model were compared with those from 100 models generated by randomly permuting the Y vector (adipose tissue proVA CAR concentrations) while keeping the X matrix intact.

A multiple‐response PLS regression (PLS2) model was additionally constructed, with adipose tissue concentrations of BCAR, ACAR, and BCRY simultaneously entered as Y variables. The same predictor preselection, standardization, VIP‐based variable selection, and internal validation procedures as described above for single‐response PLS models were applied.

All PLS analyses and validations were conducted using SIMCA Multivariate Data Analytics Solution (Version 17.0.0.24543, Umetrics, Umeå, Sweden).

## 3 Results

### 3.1 Adipose Tissue proVA CAR Concentrations

Neither sampling time (in the fasting state vs. 8 h postmeal) nor test meal type (control, vitamin E, or tomato puree) had a significant effect on adipose tissue proVA CAR concentrations (all main‐effect *p*‐values > 0.4; Tables S2A–C). Although interaction *p*‐values were relatively low for BCAR and BCRY, exploratory paired *t*‐tests (in the fasting state vs. 8 h after each meal) did not confirm any significant changes (Tables S3A–C). Therefore, we used the arithmetic mean of concentrations measured across all sampling times and test meals to represent the average adipose tissue proVA CAR concentrations (i.e., BCAR, ACAR, and BCRY) for each participant.

**FIGURE 2:**
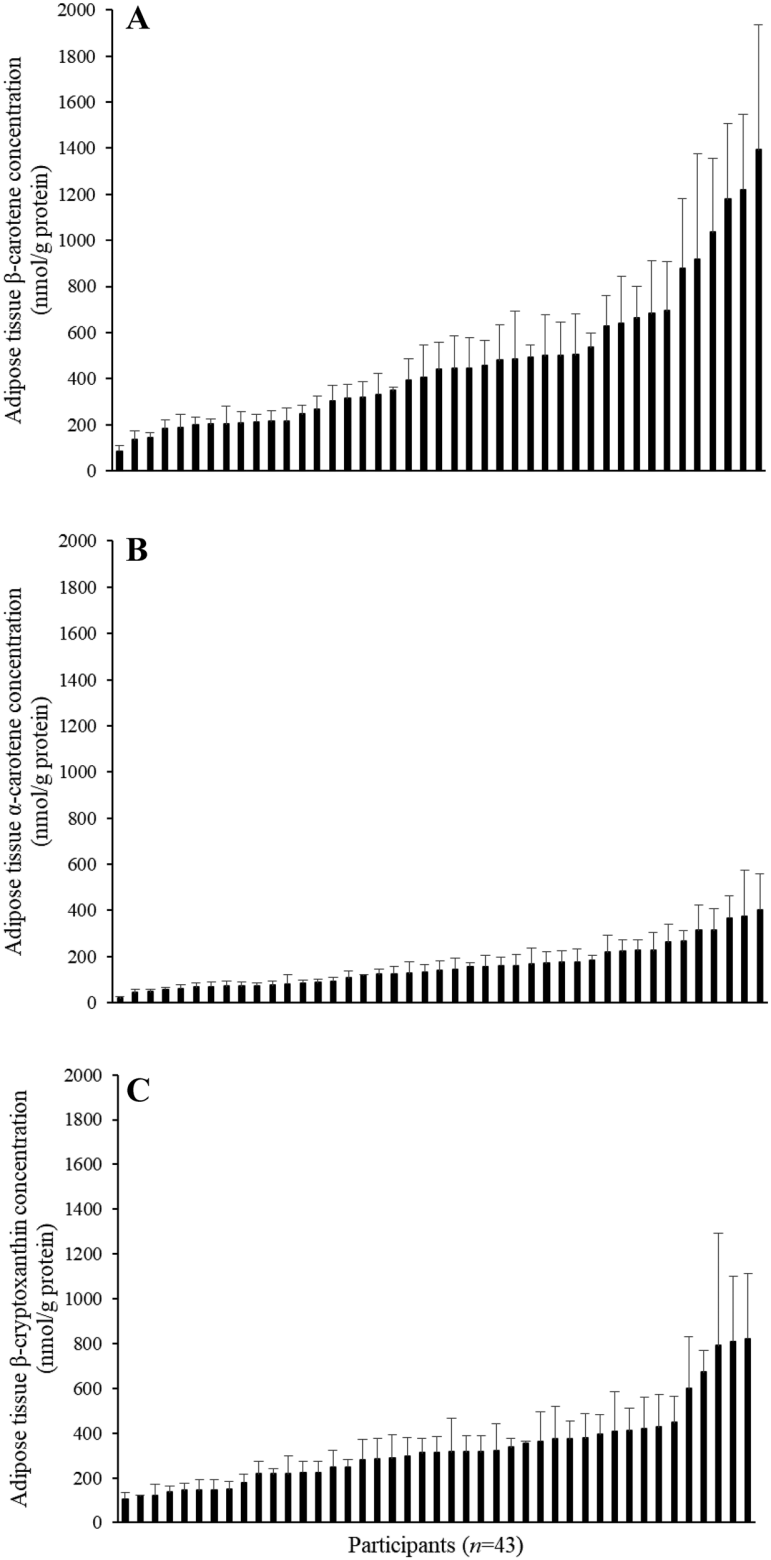
Adipose tissue provitamin A carotenoid concentrations of the participants. Adipose tissue concentrations of β‐carotene (A), α‐carotene (B), and β‐cryptoxanthin (C) were determined for each participant as the average of the concentrations measured in periumbilical adipose tissue samples. These samples were collected at two time points on three occasions: in the fasting state and 8 h after the consumption of a test meal. The reported values represent means based on measurements taken 2 to 6 times per participant and are accompanied by their corresponding SEM. A total of 43 participants were included in this analysis, and they were organized in increasing order based on their adipose tissue provitamin A carotenoid concentrations.

Adipose tissue proVA CAR concentrations are shown in Figure 2. They ranked as BCAR > BCRY > ACAR (472.3 ± 74.2, 331.6 ± 27.3, and 156.5 ± 14.4 nmol g^−1^ protein, respectively; *p* < 0.001). They exhibited high interindividual variability (CV: BCAR 66%, ACAR 61%, BCRY 54%). Fasting plasma proVA CAR concentrations ranked as in the adipose tissue, i.e. BCAR > BCRY > ACAR (0.30 ± 0.03, 0.18 ± 0.02, and 0.11 ± 0.01 µmol L^−1^, respectively; *p* < 0.001). Furthermore, adipose tissue and fasting plasma BCAR and ACAR concentrations were significantly correlated (BCAR: Pearson's *r* = 0.580 [95% CI: 0.325, 0.750], *p* = 7.1×10^−5^; ACAR: *r* = 0.507 [95% CI: 0.213, 0.710], *p* = 0.001). However, we did not find any significant correlation between adipose tissue and fasting plasma BCRY concentrations (*r* = 0.034 [95% CI: −0.294, 0.354], *p* = 0.842). Moreover, adipose tissue proVA CAR concentrations were significantly and highly correlated with each other, with the lowest correlation between BCRY and BCAR at *r* = 0.668. Correlation coefficients between adipose tissue proVA CAR concentrations and other parameters are presented in Table 2.

**TABLE 2:** Pearson's correlation coefficients between adipose tissue provitamin A carotenoid concentrations and selected anthropometric and biochemical variables.

	Adipose tissue BCAR concentration		Adipose tissue ACAR concentration		Adipose tissue BCRY concentration	
	Pearson's *r*	*p*‐value	Pearson's *r*	*p*‐value	Pearson's *r*	*p*‐value
Age, y	0.17	0.283	0.16	0.303	0.16	0.314
BMI, kg m^−2^	−0.09	0.556	−0.07	0.675	−0.03	0.847
Fasting lipid concentration, mmol L^−1^						
*Total cholesterol*	0.29	0.058	0.32	0.037^*^	0.39	0.011^*^
*LDL‐cholesterol*	0.15	0.367	0.22	0.199	0.22	0.185
*HDL‐cholesterol*	0.36	0.048	0.32	0.056	0.31	0.066
*Triglycerides*	−0.12	0.464	−0.07	0.643	0.13	0.399
BCAR concentration						
*in fasting plasma, µmol L* ^−1^	0.67	0.000^*^	0.58	0.000^*^	0.27	0.080
*in adipose tissue, nmol g* ^−1^ *protein*	—	—	0.96	0.000^*^	0.67	0.000^*^
ACAR concentration						
*in fasting plasma, µmol L* ^−1^	0.57	0.000^*^	0.51	0.001^*^	0.35	0.037^*^
*in adipose tissue, nmol g* ^−1^ *protein*	0.96	0.000^*^	—	—	0.73	0.000^*^
BCRY concentration						
*in fasting plasma, µmol L* ^−1^	0.17	0.314	0.11	0.505	0.03	0.840
*in adipose tissue, nmol g* ^−1^ *protein*	0.67	0.000^*^	0.73	0.000^*^	—	—

* Parameters were considered significant at *α* = 0.05. Abbreviations: ACAR, α‐carotene; BCAR, β‐carotene; BCRY, β‐cryptoxanthin.

### 3.2 SNPs Associated With the Interindividual Variability of Adipose Tissue proVA CAR Concentrations

**FIGURE 3:**
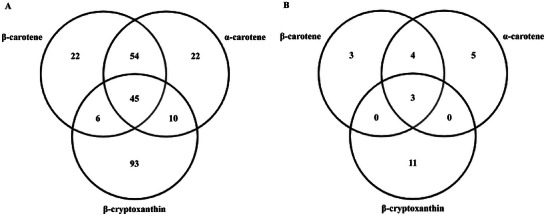
**Overlap of SNPs associated with adipose tissue provitamin A carotenoids**. Venn diagrams showing the overlap of single nucleotide polymorphisms (SNPs) associated with adipose tissue concentrations of β‐carotene, α‐carotene, and β‐cryptoxanthin. SNPs included were those significantly associated in univariate analyses (A) and in partial least squares regression models (B).

We first performed univariate association analyses between SNPs and adipose tissue proVA CAR concentrations to preselect candidate SNPs for subsequent multivariate PLS modeling (Tables S4). For BCAR (Table S4A), under the additive model, 27 SNPs within 12 genes showed significant associations, while 100 SNPs within 21 genes were significantly associated under the dominant model. For ACAR (Table S4B), under the additive model, 27 SNPs within 13 genes showed significant associations, while 104 SNPs within 20 genes were significantly associated under the dominant model. For BCRY (Table S4C), under the additive model, 26 SNPs within 9 genes showed significant associations, while 128 SNPs within 27 genes were significantly associated under the dominant model. Forty‐five SNPs were significantly associated with all three adipose tissue proVA CAR concentrations (Figure 3 **;** Table S4D).

The large majority of SNPs significantly associated with at least one proVA CAR mapped to intronic or intergenic regions, with only a minority located within exons. Among these nonexonic SNPs, approximately 10% exhibited high RegulomeDB scores (>0.7), suggesting strong regulatory potential, while 53% displayed moderate scores (0.4–0.7) and 37% low scores (<0.4). Unstandardized regression coefficients (*B*), representing the mean change in adipose tissue proVA CAR concentration (nmol g^−1^ protein) per additional copy of the minor allele (additive model) or in carriers of the minor allele (dominant model), are provided in Tables S4A–D.

### 3.3 Combinations of SNPs Associated With the Interindividual Variability of Adipose Tissue proVA CAR Concentrations

We then applied PLS regression to identify the combinations of SNPs—preselected through univariate filtering—and covariates—retained based on nonzero correlations—that best explained interindividual variability in adipose tissue proVA CAR concentrations. As shown in Tables S5, the initial models including all selected predictors (SNPs and covariates) explained a large proportion of the variance in these phenotypes (*R* ^2^ = 0.91, 0.89, and 0.87 for BCAR, ACAR, and BCRY, respectively). However, these values were inflated, as indicated by adjusted *R* ^2^ > 1, reflecting overfitting due to the high predictor‐to‐sample ratio. To obtain more stable and interpretable models, we filtered predictors based on their VIP scores. The resulting reduced models, highlighted in bold in Tables S5, achieved adjusted *R* ^2^ values of 0.69, 0.67, and 0.59 for BCAR (10 SNPs in 8 genes and fasting plasma BCAR concentrations), ACAR (12 SNPs in 8 genes and fasting plasma ACAR concentrations), and BCRY (14 SNPs in 9 genes), respectively (Tables 3A–C).

**TABLE 3:** Combination of SNPs associated with adipose tissue proVA CAR concentrations following PLS regressions.

A	BCAR	
Covariates	VIP value^b^	Regression coefficient^c^
Fasting plasma BCAR, µmol/L	1.86	335.4
Gene^a^‐ SNP		
*CXCL8 ‐* rs4694627	1.47	−64.5
*MGLL—*rs567384	1.40	110.8
*LIPC—*rs2414558	1.30	−83.8
*PTPRT—*rs4812537	1.29	−83.7
*PPARG—*rs1152002	1.27	59.1
*PPARG—*rs7618046	1.26	52.2
*BCO1 ‐* rs6564863	1.25	−80.7
*IRS1 ‐* rs7558381	1.25	−82.7
*PPARG—*rs709157	1.25	80.0
*CAPN2 ‐* rs3738383	1.22	79.4

B	ACAR	
Covariates	VIP value^b^	Regression coefficient^c^
Fasting plasma ACAR, µmol L^−1^	1.39	162.6
Gene^1^‐ SNP		
*CXCL8 –* rs4694627	1.53	−18.8
*PPARG—*rs13076933	1.33	24.0
*PPARG—*rs709157	1.3	23.5
*PTPRT—*rs4812537	1.28	−23.4
*PPARG—*rs7618046	1.28	14.9
*CAPN2 –* rs3738383	1.27	23.2
*BCO1 –* rs8060294	1.25	22.6
*CAPN2 –* rs1153947	1.24	23.2
*PPARG—*rs709158	1.24	15.5
*IRS1 –* rs7558381	1.22	−22.8
*LIPC—*rs2414558	1.21	−25.0
*MGLL—*rs7640916	1.21	−25.2

C	BCRY	
Covariates	VIP value^b^	Regression coefficient^c^
Gene^1^‐ SNP		
*PPARG—*rs1152004	1.40	45.4
*PTPRT—*rs3890324	1.36	45.5
*PTPRT—*rs1546886	1.34	31.9
*PPARG—*rs1152001	1.29	−42.0
*CAPN8 –* rs11261272	1.29	−30.8
*PPARG—*rs1151996	1.29	29.7
*IRS1 –* rs7558381	1.27	−41.9
*PTPRT—*rs7271939	1.25	53.8
*ISX—*rs5749872	1.22	44.6
*PPARG—*rs709157	1.21	38.5
*MC4R—*rs11152240	1.18	54.2
*ABCA1 –* rs2740486	1.17	−42.6
*CXCL8 –* rs4694627	1.17	−38.1
*INSIG2 –* rs4564774	1.17	53.6

D	Combined BCAR, ACAR and BCRY			
Covariates	VIP value^b^	BCAR, Regression coefficient^c^	ACAR, Regression coefficient^c^	BCRY, Regression coefficient^c^
Fasting plasma BCAR, µmol L^−1^	1.15	387.1	113.5	185.3
Fasting plasma ACAR, µmol L^−1^	1.00	774.5	227.1	370.8
Gene^1^‐ SNP				
*CXCL8 –* rs4694627	0.98	−120.1	−35.2	−57.5
*PPARG—*rs1152004	0.96	116.9	34.3	55.9
*PPARG—*rs709157	0.95	113.8	33.4	54.5
*IRS1 –* rs7558381	0.94	−116.9	−34.3	−56.0

^a^ Gene names can be found in Table S1.

^b^ Variables were ranked according to their variable importance in the projection (VIP) value, which estimates their contribution in the projection used in the PLS regression model.

^c^ Regression coefficients are for untransformed variables and represent the mean change in adipose tissue provitamin A carotenoid concentration (nmol g−1 protein) for each additional copy of the minor allele under the additive model, and in the presence of the minor allele under the dominant model.

These models were validated by cross‐validation ANOVA (Tables S5). Then, their robustness and stability were validated by three additional methods (i.e. leave‐*k*‐out cross validation, regression coefficient stability test, *R* ^2^ and adjusted *R* ^2^ after 100 random permutations) (Table S5 Figures S2, S3).

We next explored whether a single PLS regression model could simultaneously explain interindividual variability in adipose tissue concentrations of ACAR, BCAR, and BCRY. Applying the same strategy as above, a multivariate model was obtained that retained 4 SNPs located in 3 genes (*CXCL8*, *PPARG*, *IRS1*), in addition to fasting plasma ACAR and BCAR concentrations, as significant predictors (Table 3D). Together, these variables accounted for 45% of the variance in adipose tissue proVA CAR concentrations (adjusted *R* ^2^ = 0.45). Model validity and robustness were checked by cross‐validation and permutation testing (Table S5D).

## 4 Discussion

The objective of this study was to determine whether genetic variations are associated with proVA CAR concentrations in white adipose tissue, which constitutes one of their main storage sites in the human body. To this end, we conducted a secondary analysis of a randomized crossover clinical trial in which abdominal adipose tissue samples were collected from 43 healthy males, both in the fasting state and 8 h after the consumption of three different test meals. Among these meals, only the one with tomato puree provided a significant amount of a proVA CAR, namely BCAR (400 µg). However, we did not expect to observe a significant increase in adipose tissue BCAR concentration over 8 h. Indeed, El‐Sohemy et al. previously reported an adipose tissue BCAR concentration of 0.20 ± 0.18 µg g^−1^ (mean ± SD) in 344 males [26]. Assuming a mean fat mass of 14 kg/participant [50], this corresponds to a total BCAR pool of 2.8 ± 2.5 mg in adipose tissue. Even under a bioavailability estimate of 20% for BCAR from the tomato puree meal [51], this would mean that 80 µg (20% of 400 µg) of BCAR could reach the circulation. Even if all of this were deposited in adipose tissue within 8 h, it would only account for about 2.9% (80 µg of 2.8 mg) of the total BCAR pool—well within the expected range of analytical and sampling variability for adipose biopsies—and thus unlikely to be detectable or biologically meaningful. This reasoning was confirmed by the absence of significant differences (*p* > 0.05) in proVA CAR concentrations before and after the meals. We therefore treated the six measurements as technical replicates, allowing a more precise estimate of adipose tissue proVA CAR concentrations, whereas all previous studies to date have relied on single measurements [16, 18, 26].

The distribution of proVA CAR concentrations in adipose tissue observed in our study, namely BCAR > BCRY > ACAR, is consistent with that reported by Chung et al., who measured proVA CAR concentrations in three adipose depots (abdominal, gluteal, and femoral) [16]. On the other hand, El‐Sohemy et al. reported comparable BCAR and ACAR concentrations in gluteal adipose tissue, both apparently higher than that of BCRY, although no statistical comparison was performed [26]. However, since adipose tissue proVA CAR concentrations are at least partly correlated with dietary intake, these differences could reflect more variations in dietary exposure [1, 52] rather than methodological or biological factors. Nevertheless, these concentrations could also be modulated by differences in the bioavailability and metabolism of these compounds. Adipose tissue concentrations of proVA CAR exhibited high interindividual variability, with CV between 54% and 66%. This high degree of variability is quite surprising considering participants formed a relatively homogeneous group, suggesting that variability may be even greater in the general population, particularly across populations with different genetic backgrounds, as genetic variation is known to influence micronutrient metabolism [53, 54, 55]. However, these variabilities are in line with previous reports from El‐Sohemy et al. (ACAR, 75%; BCAR, 90%; BCRY, 75%; reported CV are for gluteal abdominal tissue in males) [26] and Chung et al. (ACAR, 132%; BCAR, 97%; BCRY, 56%; reported CV are for abdominal adipose tissue in both males and females) [16].

We then aimed to identify genetic determinants contributing to these variabilities, as proVA CAR concentrations in adipose tissue are partly governed by protein‐mediated processes and therefore by genes, integrating as well available demographic and biochemical information. For each of the three proVA CAR, we identified a combination of SNPs significantly associated with their adipose tissue concentration. Notably, three SNPs were common to all three models: rs4694627 in *CXCL8*, rs7558381 in *IRS1*, and rs709157 in *PPARG* (Figure 3). It was therefore not surprising that these SNPs were retained in the final multiple PLS regression model, together with another SNP in *PPARG* (rs1152004). This SNP was significantly associated with adipose tissue BCAR and ACAR concentrations in univariate analyses (*p* = 0.0039 and 0.0032, respectively), but it was not retained in the final PLS regression models built separately for each proVA CAR because its VIP value was below the inclusion threshold. Moreover, the selected PLS regression models for BCAR and ACAR shared seven SNPs (out of 10 and 12 SNPs, respectively), whereas only three of these SNPs were also included in the selected model for BCRY. Combined with the strong correlation observed between adipose tissue BCAR and ACAR concentrations (*r* = 0.96), this suggests that these two CAR are likely to follow comparable transport and metabolic processes in adipose tissue. Interestingly, fasting plasma BCAR and ACAR concentrations were strong predictors of their respective adipose tissue concentrations, as indicated by their high VIP values. This aligns with the significant correlations observed between fasting plasma and adipose tissue concentrations for these two CAR (*r* > 0.5). In contrast, adipose tissue BCRY concentrations exhibited distinct behavior compared to BCAR and ACAR, with only three shared SNPs in the selected PLS regression model, no significant correlation with fasting plasma BCRY concentration, and only moderate correlations with adipose tissue BCAR and ACAR concentrations (*r* = 0.67 and 0.73 with BCAR and ACAR, respectively). Finally, we did not find any association between BMI and adipose tissue proVA CAR concentrations, contrary to previous reports [16, 23]. However, this may be explained by the relatively homogeneous BMI of the participants, with a CV of only 8.6%.

The results showed that 26 unique SNPs across 13 genes were associated with adipose tissue concentrations of the three proVA CAR in the selected PLS regression models. As it was not possible to propose mechanistic hypotheses for all these associations, we focused on the three genes identified in the multiple PLS regression model—*CXCL8*, *IRS1*, and *PPARG*—and on two additional genes known to play key roles in CAR metabolism, namely *BCO1* and *ISX*. The genetic variant with the strongest influence on the combined phenotypes was rs4694627, located in an intergenic region near *CXCL8*. It is therefore likely to be in LD with a functional SNP within this gene that remains to be identified. *CXCL8* encodes chemokine (C–X–C motif) ligand 8, commonly referred to as interleukin‐8 (IL‐8), a key mediator of immune cell recruitment and local inflammatory responses [56]. Previous studies have shown that CAR can modulate inflammatory pathways [24, 57], including IL‐8 expression [56], while inflammation itself may influence CAR metabolism and tissue distribution [18]. These findings raise the possibility that interindividual differences in IL‐8 concentrations—potentially influenced by *CXCL8* genetic variation—could contribute to variability in adipose tissue CAR concentrations, either through local oxidative processes or via altered lipid/CAR trafficking in inflamed adipose depots. Notably, the specific SNP reported here was also associated with adipose tissue zeaxanthin concentration in the same cohort [58]. Furthermore, three additional SNPs in *CXCL8* were associated with BCAR bioavailability in this cohort [32], suggesting that the observed link with adipose tissue CAR could also result indirectly from systemic effects on absorption, transport, or metabolism. Although these interpretations remain speculative, they highlight IL‐8 as a potential molecular link between inflammation, CAR metabolism, and CAR storage in adipose tissue, warranting further investigation in targeted studies.

The second and third SNPs that most impacted the concentrations of the three proVA CAR in adipose tissue were two SNPs in or near the *PPARG* gene, namely rs1152004 (intergenic) and rs709157 (intronic). *PPARG* encodes peroxisome proliferator‐activated receptor γ (PPARγ), an orphan nuclear receptor that acts as a lipid sensor, with its expression and activity strongly influenced by nutritional status [59]. *PPARG* is highly expressed in adipose tissue, where it regulates adiposity by controlling adipocyte differentiation and lipid metabolism. This transcription factor also modulates numerous genes and metabolic pathways, including those involved in lipid droplet formation [60]. Given that CAR are highly lipophilic molecules stored within adipocytes, primarily in the lipid droplet, genetic variants in genes affecting adipocyte differentiation, lipid droplet biogenesis, or lipid turnover may directly influence CAR accumulation and retention. Beyond rs709157, which was associated with the adipose tissue concentrations of all three proVA CAR in the final PLS regression models, seven additional *PPARG* SNPs were linked to the concentration of at least one proVA CAR. In the same cohort, rs1151996 and rs13076933 were associated with adipose tissue retinol [61], rs13076933 and rs709158 with α‐tocopherol [35], and rs709158 and rs1152004 with lutein and zeaxanthin [58]. Altogether, these findings underscore the pivotal role of PPARG in regulating adipose tissue concentrations of several fat‐soluble micronutrients.

The fourth SNP that most impacted the concentrations of the three proVA CAR in adipose tissue was an intergenic SNP near the *IRS1* gene, suggesting that a functional SNP in this gene, and in LD with this intergenic SNP, was involved. *IRS1* encodes insulin receptor substrate 1, a key mediator of insulin signal transduction. Insulin has been shown to inhibit chylomicron production in healthy males [62] and to regulate lipoprotein lipase activity in adipose tissue — the enzyme responsible for chylomicron triglyceride hydrolysis and a ligand involved in receptor‐mediated chylomicron clearance from circulation [63]. *IRS1* was selected as a candidate gene in this study because we previously demonstrated, in the same cohort, that SNPs in this gene influence the postprandial chylomicron triglyceride response [37]. Since chylomicrons are the main blood carriers of newly absorbed CAR, we hypothesized that *IRS1* variants could modulate CAR bioavailability, which we confirmed for lutein [34]. Interestingly, the same SNP was also associated with adipose tissue lycopene concentrations in this cohort [64], further supporting a role of insulin signaling in CAR metabolism. Notably, Harari et al. reported negative correlations between adipose tissue CAR concentrations and insulin resistance [18]. Although these findings were discussed in terms of the potential insulin‐sensitizing effects of CAR, the reverse causal direction — i.e., that insulin resistance could alter CAR metabolism — cannot be excluded. Interestingly, the intergenic SNP rs7558381 identified in our study is in strong LD with rs2943650 (*R* ^2^ ≈ 1.0) within the *IRS1* locus in European populations (1000 Genomes CEU, LDlink). The rs2943650 variant has been associated with alterations in adipose tissue morphology, insulin sensitivity, and circulating lipid concentrations. In particular, rs2943650 was associated with decreased IRS1 expression in subcutaneous adipose tissue [65] and in a GWAS, it was also associated with increased visceral to subcutaneous fat ratio, insulin resistance, and dyslipidemia, suggesting that this SNP affects adipocyte function and lipid metabolism [66]. Given this tight LD, the association observed here between rs7558381 and adipose tissue proVA CAR concentrations may reflect a shared genetic mechanism linking *IRS1*‐related insulin signaling, adipose lipid handling, and CAR storage.

Among the other SNPs involved, two were located in key genes involved in CAR metabolism, i.e., *BCO1* and *ISX*. It is indeed noteworthy that rs6564863 and rs8060294 in *BCO1* were associated with adipose tissue concentrations of BCAR and ACAR, respectively, and that rs5749872 in *ISX* was associated with BCRY concentration. *BCO1* encodes β‐carotene oxygenase 1, the enzyme responsible for the central cleavage of proVA CAR to produce, after an intermediate step of retinal reduction, retinol. *BCO1* is primarily expressed in jejunal enterocytes [67], where the majority (>70%) of proVA CAR conversion occurs [68] but this conversion also occurs in the adipose tissue [20], which could explain the present association. Moreover, several SNPs in or near *BCO1* have been associated with circulating concentrations of proVA CAR and retinol, as well as proVA CAR bioconversion [69, 70, 71, 72, 73, 74]. Thus, it is also plausible that SNPs in *BCO1*, by altering the circulating pool of proVA CAR—which serves as the source for adipose tissue proVA CAR—could in turn modulate adipose tissue proVA CAR concentrations. Concerning *ISX*, it encodes the intestine‐specific homeobox transcription factor, which is specifically expressed in the intestine and represses both *BCO1* and *SCARB1* [75]. *SCARB1* encodes SR‐B1, a membrane protein involved in lipid transport, including that of lipophilic micronutrients [76]. Our group has demonstrated that SR‐B1 mediates the enterocyte uptake of proVA CAR and that SNPs in *SCARB1* are associated with circulating proVA CAR concentrations [77]. Therefore, the association of rs5749872 in *ISX* with adipose tissue BCRY concentration may be attributable to its effects on *BCO1* and/or *SCARB1*, potentially influencing BCRY bioavailability. Notably, rs5749872 was also associated with adipose tissue lycopene concentration in the same cohort [64].

This study has several limitations. First, because no dietary survey was conducted, we could not assess dietary intakes of proVA CAR. Chung et al. reported Pearson's *r* values of 0.43, 0.24, and 0.47 between abdominal adipose tissue concentrations of BCAR, ACAR, and BCRY and their dietary intakes, respectively (*n* = 25; 12 females and 13 males) [16], whereas El‐Sohemy et al. observed weaker correlations between gluteal adipose tissue proVA CAR concentrations and their dietary intakes (Spearman's *ρ* values of 0.07, 0.04, and 0.23 for BCAR, ACAR, and BCRY, respectively; *n* = 344 males) [26]. Although we included plasma proVA CAR concentrations among the explanatory variables—which exhibit partial correlation with dietary intakes [16, 26, 78]—only BCAR and ACAR plasma concentrations were retained in the final PLS models for their respective adipose tissue concentrations. Including dietary intake data might therefore have further increased the proportion of variance explained. Adipose tissue proVA CAR concentrations are complex phenotypes influenced by both the uptake of circulating proVA CAR and their metabolism within the tissue. Consequently, many genes are likely involved, and our candidate gene approach may likely have missed genes that have not yet been implicated in the metabolism of these compounds in adipose tissue. Furthermore, we focused exclusively on one type of genetic variation, namely SNPs. Although SNPs are the most frequent genetic variants in the genome, other forms of variation—such as structural variants—could also contribute to interindividual variability in adipose tissue proVA CAR concentrations [79]. Finally, our sample included only 43 healthy Caucasian adult males. Females have been shown to exhibit higher adipose tissue CAR concentrations than males [16], partly explained by typically higher dietary CAR intakes, but sex‐related differences in proVA CAR metabolism cannot be excluded. Moreover, the relatively small sample size (*n* = 43) may have limited the statistical power to detect additional associations. Therefore, the associations reported here should be validated in larger cohorts and extended to other populations, including females and diverse ethnic groups. Taken together, these limitations indicate that the present work should be considered exploratory.

Nevertheless, this study is, to our knowledge, the first to identify genes and SNPs associated with proVA CAR concentrations in adipose tissue, one of the main storage sites of these compounds in the human body. It thus provides a foundation for future research aimed at identifying the determinants of proVA CAR status. In the longer term, these findings may contribute to the development of personalized nutritional strategies aimed at improving proVA CAR status according to individual genetic backgrounds, although this will require confirmation in larger and more diverse populations. Such insights could help identify individuals at risk of low adipose tissue proVA CAR concentrations, potentially increasing their susceptibility to oxidative stress–related conditions such as cardiovascular disease, diabetes, obesity, and certain cancers [21, 80, 81].

## Author Contributions

The authors’ responsibilities were as follows. **Mark Pretzel Zumaraga**: choice of candidate genes, statistical analysis. **Patrick Borel**: conceptualization, methodology, funding acquisition, project administration, supervision, choice of candidate genes, writing – review & editing. **Charles Desmarchelier**: methodology, choice of candidate genes, statistical analysis, supervision, writing – original draft. All authors have read and approved the final manuscript.

## Funding

This study was supported by the European Community's Sixth Framework Program. The funding was attributed to the Lycocard project (no. 016213), which was an Integrated Project within the framework of the “Food Quality and Safety” program.

## Conflicts of Interest

The authors declare no conflict of interest.

## Supporting information




**Supporting File 1**: mnfr70557‐sup‐0001‐SuppMat.docx.

**Supporting File 2**: mnfr70557‐sup‐0002‐TableS4.xlsx.

## Data Availability

Although data sharing was not included in the original ethics approval, de‐identified individual‐level data or aggregated datasets may be made available upon reasonable request, subject to approval by the relevant ethics committee and in accordance with applicable data protection regulations.
