# Supplementary material for: Genetic Determinants of Interindividual Differences in Provitamin A Carotenoid Concentrations in Human Adipose Tissue
Source: Mol Nutr Food Res. 2026 Jul 27;70(14):e70557. doi: 10.1002/mnfr.70557 (PMC13402975; doi:10.1002/mnfr.70557)
Supplement: Supplementary file 1 — Supporting File 1: mnfr70557‐sup‐0001‐SuppMat.docx. [file MNFR-70-e70557-s001.docx]

**Supporting Tables, Figures and Methods**

**Supplemental Table S1.** Candidate genes selected.

| Gene Name | Gene symbol | References |
| --- | --- | --- |
| Genes that play, or are assumed to play, a role in proVA CAR or triglyceride metabolism in adipose tissue | | |
| ATP binding cassette subfamily A member 1 | *ABCA1* | [1] |
| β-carotene 15,15’oxygenase-1 | *BCO1* | [2] |
| β-carotene 9,10’ oxygenase-2 | *BCO2* | [3] |
| **Cluster of Differentiation** 36 | *CD36* | [4] |
| ELOVL fatty acid elongase 5 | *ELOVL5* | [5] |
| GRAM domain containing 1A | *GRAMD1A* | [6, 7] |
| GRAM domain containing 1C | *GRAMD1C* | [6, 7] |
| Low density lipoprotein receptor | *LDLR* | [8] |
| Lipase E | *LIPE* | [9] |
| Lipoprotein lipase | *LPL* | [10] |
| Monoglyceride lipase | *MGLL* | [11] |
| Polycystic kidney disease 1-like 2 | *PKD1L2* | [12] |
| Patatin-like Phospholipase Domain-containing 2 | *PNPLA2* | [9] |
| Peroxisome proliferator activated receptor gamma | *PPARG* | [13] |
| Scavenger receptor class B member 1 | *SCARB1* | [14] |
| Genes with GWAS-reported SNPs associated with circulating proVA CAR concentrations | | |
| Calpain 2 | *CAPN2* | [15] |
| Calpain 8 | *CAPN8* | [15] |
| Protein kinase C epsilon | *PRKCE* | [15] |
| Protein tyrosine phosphatase receptor type T | *PTPRT* | [16] |
| Genes whose SNPs have been associated with postprandial chylomicron CAR or triglyceride responses in the same cohort | | |
| ATP binding cassette subfamily G member 2 | *ABCG1* | [12] |
| ATP binding cassette subfamily G member 5 | *ABCG2* | [12] |
| Apolipoprotein A1 | *APOA1* | [17] |
| Apolipoprotein A3 | *APOA3* | [17] |
| Apolipoprotein A4 | *APOA4* | [17] |
| Apolipoprotein A5 | *APOA5* | [17] |
| Apolipoprotein B | *APOB* | - [12] |
| COBL-like 1 | *COBLL1* | - [17] |
| Chemokine (C-X-C motif) ligand 8 | *CXCL8* | - [12] |
| ELOVL fatty acid elongase 2 | *ELOVL2* | - [12] |
| Fatty acid desaturase 1 | *FADS1* | [17] |
| Fatty acid desaturase 2 | *FADS2* | [17] |
| Fatty acid desaturase 3 | *FADS3* | [17] |
| Insulin induced gene 2 | *INSIG2* | [12] |
| Insulin receptor substrate 1 | *IRS1* | [17] |
| Intestine specific homebox | *ISX* | [12] |
| Lipase, hepatic | *LIPC* | [12] |
| Melanocortin 4 receptor | *MC4R* | [17] |
| Microsomal triglyceride transfer protein | *MTTP* | [12] |
| Niemann-Pick disease, type C1, gene-like 1 | *NPC1L1* | - [12] |
| Pancreatic lipase | *PNLIP* | - [12] |
| Retinal pigment epithelium-specific protein 65kDa | *RPE65* | - [12] |
| Solute carrier family 27, member 6 | *SLC27A6* | [12] |
| Superoxide dismutase 2, mitochondrial | *SOD2* | [12] |
| Transcription factor 7 like 2 | *TCF7L2* | [12] |

**Supplemental Tables S2.** Effect of time and type of meal on adipose tissue proVA CAR concentrations.

1. **BCAR**

| Parameters^a^ | Numerator df | Denominator df | F | Sig.^b^ |
| --- | --- | --- | --- | --- |
| Intercept | 1 | 43.1 | 96.8 | 0.000 |
| Time (Fasting vs 8 h) | 1 | 36.9 | 0.7 | 0.417 |
| Type of Meal (Control vs vitamin E vs Tomato Puree) | 2 | 38.5 | 0.6 | 0.557 |
| Time * Type of Meal | 2 | 39.3 | 2.3 | 0.118 |

**^a^**Unstructured mixed model. Adipose tissue BCAR concentrations measured at fast and 8 h after consumption of the three test meals was analyzed with linear mixed models, using a full factorial design with Type of Meal (control, vitamin E and tomato puree) and Time (fasting and 8 h post-meal) as fixed within-subject variables and participant as the random variable. Of the five linear mixed models tested, the unstructured model was selected based on Akaike’s Information Criterion [34].

**^b^** Parameters were considered significant at 0.05 level.

1. **ACAR**

| Parameters^a^ | Numerator df | Denominator df | F | Sig.^b^ |
| --- | --- | --- | --- | --- |
| Intercept | 1 | 175.0 | 187.4 | 0.000 |
| Time (Fasting vs 8 h) | 1 | 175.0 | 0.6 | 0.436 |
| Type of Meal (Control vs vitamin E vs Tomato Puree) | 2 | 111.0 | 0.7 | 0.516 |
| Time * Type of Meal | 2 | 111.0 | 1.4 | 0.263 |

**^a^**Diagonal mixed model. Adipose tissue ACAR concentrations measured at fast and 8 h after consumption of the three test meals was analyzed with linear mixed models, using a full factorial design with Type of Meal (control, vitamin E and tomato puree) and Time (fasting and 8 h post-meal) as fixed within-subject variables and participant as the random variable. Of the five linear mixed models tested, the diagonal model was selected based on Akaike’s Information Criterion [34].

**^b^** Parameters were considered significant at 0.05 level.

1. **BCRY**

| Parameters^a^ | Numerator df | Denominator df | F | Sig.^b^ |
| --- | --- | --- | --- | --- |
| Intercept | 1 | 35.9 | 175.4 | 0.000 |
| Time (Fasting vs 8 h) | 1 | 36.2 | 0.2 | 0.662 |
| Type of Meal (Control vs vitamin E vs Tomato Puree) | 2 | 37.5 | 0.9 | 0.398 |
| Time * Type of Meal | 2 | 36.6 | 2.1 | 0.131 |

**^a^**Unstructured mixed model. Adipose tissue BCRY concentrations measured at fast and 8 h after consumption of the three test meals was analyzed with linear mixed models, using a full factorial design with Type of Meal (control, vitamin E and tomato puree) and Time (fasting and 8 h post-meal) as fixed within-subject variables and participant as the random variable. Of the five linear mixed models tested, the unstructured model was selected based on Akaike’s Information Criterion [34].

**^b^** Parameters were considered significant at 0.05 level.

**Supplemental Tables S3.** Effect of time within each meal on adipose tissue proVA CAR concentrations.

1. **BCAR**

| **Type of Meal** | **Paired Differences^a^** | | | | | **t** | **df** | **Sig.^b^** |
| --- | --- | --- | --- | --- | --- | --- | --- | --- |
|  | **Mean** | **SD** | **SEM** | **95% CI** | |  |  |  |
|  |  |  |  | **Lower** | **Upper** |  |  |  |
| Control Meal | -56.5 | 627.0 | 107.5 | -275.3 | 162.2 | -0.526 | 33 | 0.603 |
| α-TOC Meal | 6.8 | 626.6 | 110.8 | -219.1 | 232.7 | 0.061 | 31 | 0.951 |
| Tomato Puree Meal | 206.6 | 612.0 | 102.0 | -0.4 | 413.7 | 2.026 | 35 | 0.050 |

^a^The paired differences of adipose tissue BCAR concentrations between fasting and 8 h after consumption of the test meals are displayed (*n* = 43).

^b^Two-way significance test was performed. A *p*-value less than 0.05 comparing adipose tissue BCAR concentration before and after intake of each test meal was considered significant.

1. **ACAR**

| **Type of Meal** | **Paired Differences^a^** | | | | | **t** | **df** | **Sig.^b^** |
| --- | --- | --- | --- | --- | --- | --- | --- | --- |
|  | **Mean** | **SD** | **SEM** | **95% CI** | |  |  |  |
|  |  |  |  | **Lower** | **Upper** |  |  |  |
| Control Meal | -16.6 | 219.9 | 37.7 | -93.3 | 60.2 | -0.439 | 33 | 0.663 |
| α-TOC Meal | 7.7 | 190.1 | 33.6 | -60.8 | 76.2 | 0.229 | 31 | 0.820 |
| Tomato Puree Meal | 69.3 | 207.6 | 34.6 | -0.9 | 139.6 | 2.003 | 35 | 0.053 |

^a^The paired differences of adipose tissue ACAR concentrations between fasting and 8 h after consumption of the test meals are displayed (*n* = 43).

^b^Two-way significance test was performed. A *p*-value less than 0.05 comparing adipose tissue ACAR concentration before and after intake of each test meal was considered significant.

1. **BCRY**

| **Type of Meal** | **Paired Differences^a^** | | | | | **t** | **df** | **Sig.^b^** |
| --- | --- | --- | --- | --- | --- | --- | --- | --- |
|  | **Mean** | **SD** | **SEM** | **95% CI** | |  |  |  |
|  |  |  |  | **Lower** | **Upper** |  |  |  |
| Control Meal | -40.4 | 306.1 | 52.5 | -147. | 66.5 | -0.769 | 33 | 0.448 |
| α-TOC Meal | 4.0 | 349.3 | 61.7 | -121.9 | 130.0 | 0.065 | 31 | 0.948 |
| Tomato Puree Meal | 86.2 | 356.4 | 59.4 | -34.4 | 206.8 | 1.451 | 35 | 0.156 |

^a^The paired differences of adipose tissue BCRY concentrations between fasting and 8 h after consumption of the test meal are displayed (*n* = 43).

^b^Two-way significance test was performed. A *p*-value less than 0.05 comparing adipose tissue BCRY concentration before and after intake of each test meal was considered significant.

**Supplementary Table S4A-S4D.** SNPs significantly associated with adipose tissue provitamin A carotenoid (S4A: BCAR; S4B: ACAR; S4C: BCRY; S4D: Common) concentrations following univariate analysis (Please refer to Supplemental Table S4.xlsx file)

**Supplemental Tables S5.** Characteristics of the partial least squares regression models generated.^a^

1. **BCAR**

| **Number of predictors** | ***R*^2^** | **Adjusted *R*^2^** | ***R*² after 100 permutations^b^** | ***R*² after cross-validation^b^** | **Cross-validation-**  **ANOVA *p*-value^c^** |
| --- | --- | --- | --- | --- | --- |
| 128 (including fasting plasma BCAR) | 0.91 | 1.04 | 0.55 | 0.83 | 7.01 x 10^-16^ |
| 39 | 0.82 | -1.51 | 0.31 | 0.75 | 9.03 x 10^-13^ |
| 32 | 0.83 | 0.27 | 0.31 | 0.76 | 4.91 x 10^-13^ |
| 27 | 0.82 | 0.48 | 0.28 | 0.75 | 8.74 x 10^-13^ |
| 22 | 0.84 | 0.65 | 0.24 | 0.79 | 3.60 x 10^-14^ |
| 19 | 0.81 | 0.66 | 0.23 | 0.76 | 4.79 x 10^-13^ |
| 17 | 0.81 | 0.68 | 0.22 | 0.75 | 7.71 x 10^-13^ |
| 13 | 0.78 | 0.68 | 0.18 | 0.73 | 5.26 x 10^-12^ |
| **11** | **0.77** | **0.69** | **0.16** | **0.72** | **6.42 x 10^-12^** |
| 10 | 0.75 | 0.67 | 0.16 | 0.69 | 5.52 x 10^-11^ |
| 8 | 0.73 | 0.67 | 0.13 | 0.67 | 2.17 x 10^-10^ |
| 7 | 0.73 | 0.67 | 0.13 | 0.67 | 3.00 x 10^-10^ |
| 5 | 0.67 | 0.62 | 0.09 | 0.62 | 4.91 x 10^-9^ |
| 3 | 0.56 | 0.52 | 0.06 | 0.48 | - 1. x 10^-6^ |

1. **ACAR**

| **Number of predictors** | ***R*^2^** | **Adjusted *R*^2^** | ***R*² after 100 permutations^b^** | ***R*² after cross-validation^b^** | **Cross-validation-**  **ANOVA *p*-value^c^** |
| --- | --- | --- | --- | --- | --- |
| 133 (including fasting plasma ACAR, total plasma CHOL) | 0.89 | 1.05 | 0.56 | 0.82 | 8.27 x 10^-16^ |
| 41 | 0.84 | -5.76 | 0.33 | 0.79 | 4.63 x 10^-14^ |
| 26 | 0.75 | 0.35 | 0.22 | 0.70 | 4.94 x 10^-11^ |
| 22 | 0.75 | 0.47 | 0.21 | 0.70 | 4.59 x 10^-11^ |
| 20 | 0.75 | 0.52 | 0.22 | 0.69 | 5.72 x 10^-11^ |
| 17 | 0.74 | 0.57 | 0.20 | 0.69 | 7.70 x 10^-11^ |
| 15 | 0.74 | 0.59 | 0.19 | 0.68 | 1.14 x 10^-10^ |
| 14 | 0.74 | 0.62 | 0.19 | 0.69 | 6.35 x 10^-11^ |
| **13** | **0.77** | **0.67** | **0.18** | **0.72** | **8.01 x 10^-12^** |
| 10 | 0.71 | 0.61 | 0.16 | 0.65 | 7.01 x 10^-10^ |
| 9 | 0.67 | 0.58 | 0.14 | 0.60 | 5.85 x 10^-9^ |
| 7 | 0.60 | 0.52 | 0.11 | 0.55 | 1.04 x 10^-7^ |
| 5 | 0.59 | 0.53 | 0.09 | 0.54 | 1.87 x 10^-7^ |
| 3 | 0.56 | 0.53 | 0.06 | 0.53 | 2.37 x 10^-7^ |

1. **BCRY**

| **Number of predictors** | ***R*^2^** | **Adjusted *R*^2^** | ***R*² after 100 permutations^b^** | ***R*² after cross-validation^b^** | **Cross-validation-**  **ANOVA *p*-value^c^** |
| --- | --- | --- | --- | --- | --- |
| 155 (including total plasma CHOL) | 0.87 | 1.05 | 0.55 | 0.80 | 7.03 x 10^-15^ |
| 38 | 0.76 | -1.57 | 0.32 | 0.68 | 1.06 x 10^-10^ |
| 21 | 0.69 | 0.37 | 0.23 | 0.62 | 4.08 x 10^-9^ |
| 19 | 0.69 | 0.44 | 0.22 | 0.63 | 2.53 x 10^-9^ |
| 16 | 0.70 | 0.51 | 0.20 | 0.64 | 1.68 x 10^-9^ |
| 15 | 0.70 | 0.53 | 0.22 | 0.64 | 1.80 x 10^-9^ |
| **14** | **0.72** | **0.59** | **0.19** | **0.67** | **3.21 x 10^-10^** |
| 13 | 0.70 | 0.56 | 0.19 | 0.64 | 1.24 x 10^-9^ |
| 9 | 0.62 | 0.51 | 0.14 | 0.56 | 7.23 x 10^-8^ |
| 8 | 0.59 | 0.49 | 0.13 | 0.54 | 1.78 x 10^-7^ |
| 6 | 0.53 | 0.45 | 0.10 | 0.49 | 1.56 x 10^-6^ |
| 4 | 0.45 | 0.39 | 0.07 | 0.42 | 1.86 x 10^-5^ |
| 2 | 0.33 | 0.30 | 0.04 | 0.31 | 1.00 x 10^-3^ |

1. **Combined (BCAR, ACAR and BCRY) – Multi Y PLS regression model**

| **Number of predictors** | ***R*^2^** | **Adjusted *R*^2^** | ***R*² after 100 permutations^b^** | ***R*² after cross-validation^b^** | **Cross-validation-ANOVA *p*-value^c^** |
| --- | --- | --- | --- | --- | --- |
| 268 (including fasting BCAR, ACAR, BCRY, total cholesterol) | 0.77 | 1.04 | 0.60 | 0.67 | 4.06 x 10^-9^ |
| 33 | 0.64 | -0.68 | 0.22 | 0.57 | 8.17 x 10^-9^ |
| 4 | 0.62 | 0.11 | 0.18 | 0.56 | 7.80 x 10^-9^ |
| 17 | 0.55 | 0.25 | 0.13 | 0.50 | 1.79 x 10^-7^ |
| 16 | 0.56 | 0.29 | 0.13 | 0.50 | 1.12 x 10^-7^ |
| 15 | 0.56 | 0.31 | 0.13 | 0.50 | 1.43 x 10^-7^ |
| 14 | 0.54 | 0.31 | 0.11 | 0.48 | 6.42 x 10^-7^ |
| 13 | 0.51 | 0.29 | 0.11 | 0.45 | 2.77 x 10^-6^ |
| 9 | 0.54 | 0.42 | 0.10 | 0.50 | 1.02 x 10^-7^ |
| 8 | 0.53 | 0.41 | 0.10 | 0.48 | 2.24 x 10^-7^ |
| **6** | **0.53** | **0.45** | **0.10** | **0.49** | **8.09 x 10^-8^** |
| 4 | 0.46 | 0.40 | 0.07 | 0.42 | 5.72 x 10^-7^ |
| 2 | 0.29 | 0.25 | 0.03 | 0.25 | 5.65 x 10^-9^ |

^a^The selected model is highlighted in bold font. All models had one component.

^b^See **Supplemental Figure S3** for further explanation of the procedure.

^c^See [18].

Abbreviations: BCAR – β-carotene ; ACAR – α-carotene; BCRY – β-cryptoxanthin.

**Supplemental Figure S1.** Candidate SNP selection flowchart.

SNPs not respecting HWE (chi-squared test, *p*<0.05), SNPs with a single genotype and SNPs with a call rate <95% were removed

1142 SNPs removed

Tag SNPs selection and removal of SNPs in linkage disequilibrium (*R*²>0.8)

629 SNPs removed

447 SNPs removed

2052 SNPs removed

**Supplemental Information:** additional validations of the partial least squares regression models.

1. *Leave-*k*-out* cross-validation

The leave *k*-out validation procedure was based on [19]. In summary, we tested our partial least squares (PLS) regression models by randomly excluding k participants (k = {1, 2, 3, 4}) from the original dataset, thereby creating training datasets. The excluded k participants were then reintroduced to evaluate whether the models, built without their data, could accurately predict their adipose tissue proVA CAR concentration. This process was repeated multiple times to ensure each participant was excluded once (i.e., 43 times for k = 1, 21 times for k = 2, 14 times for k = 3, and 10 times for k = 4).

The *R*² of the simple linear regression between the predicted and the measured adipose tissue proVA CAR concentration for each *k* are shown in **Supplemental Table S3**. The percentage of error remained relatively robust, even when up to 4 participants were left out of the models, suggesting that the PLS regression models were relatively stable.

**Supplemental Table S6.** Average relative prediction error following the leave-*k*-out procedure.

| **% Error** | **Number of participants left out** | | | | |
| --- | --- | --- | --- | --- | --- |
|  | **0** | **1** | **2** | **3** | **4** |
| BCAR | 28.7 | 32.1 | 31.5 | 32.0 | 30.5 |
| ACAR | 30.0 | 33.6 | 34.1 | 34.5 | 35.7 |
| BCRY | 25.5 | 28.7 | 28.9 | 30.2 | 31.5 |
| Combined multi-Y PLS model^a^ |  |  |  |  |  |
| BCAR | 35.9 | 39.0 | 39.6 | 38.0 | 37.8 |
| ACAR | 40.8 | 44.4 | 45.1 | 43.6 | 41.9 |
| BCRY | 36.8 | 39.2 | 40.0 | 39.2 | 40.0 |

^a^A combined multi-Y partial least squares regression model was also developed to simultaneously account for the interrelationships among the three adipose tissue proVA CAR concentrations.

Abbreviations: BCAR – β-carotene ; ACAR – α-carotene; BCRY – β-cryptoxanthin.

*Regression coefficient stability testing following the leave-*k*-out procedure*

We checked that the regression coefficients of the SNPs from the selected models (**Table 3**) remained unchanged (*p*>0.05; ANOVA) following the leave-*k*-out procedure described above. **Supplemental Figures S2** show good stability of the regression coefficients with this validation.


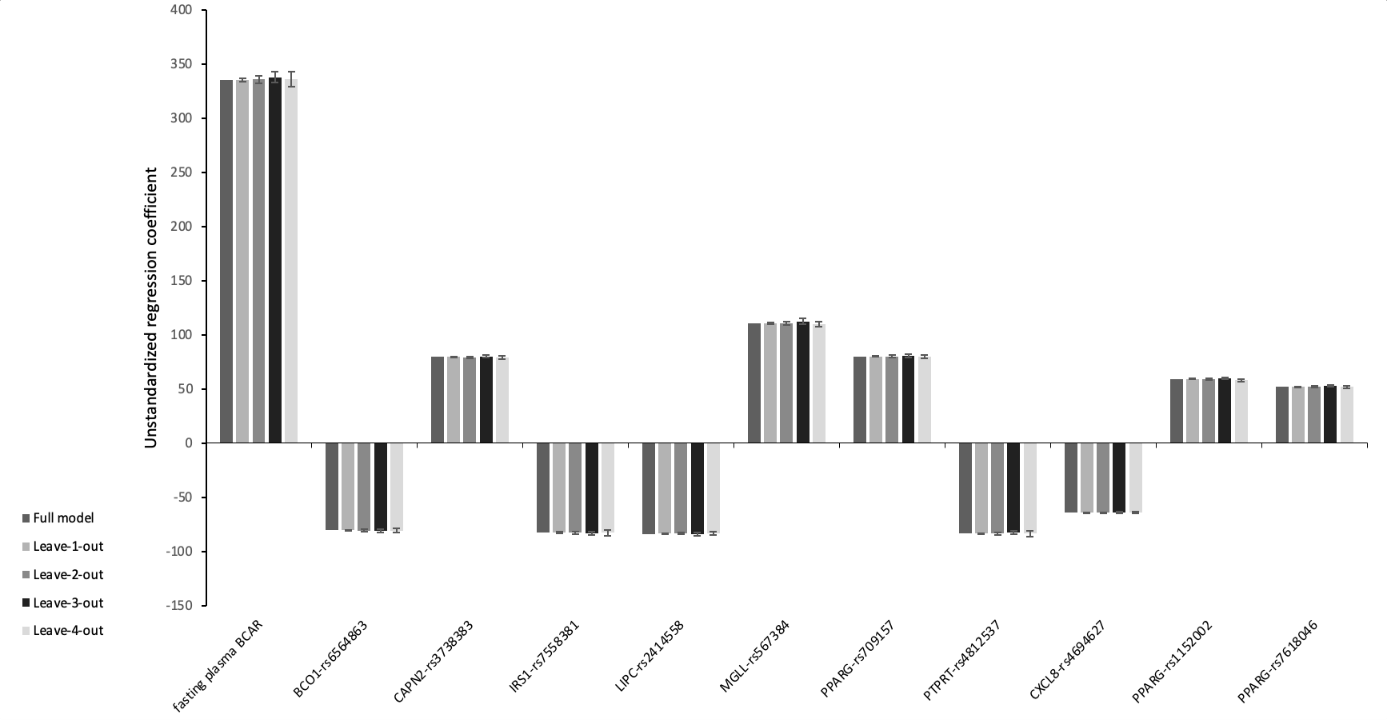


1. **BCAR**


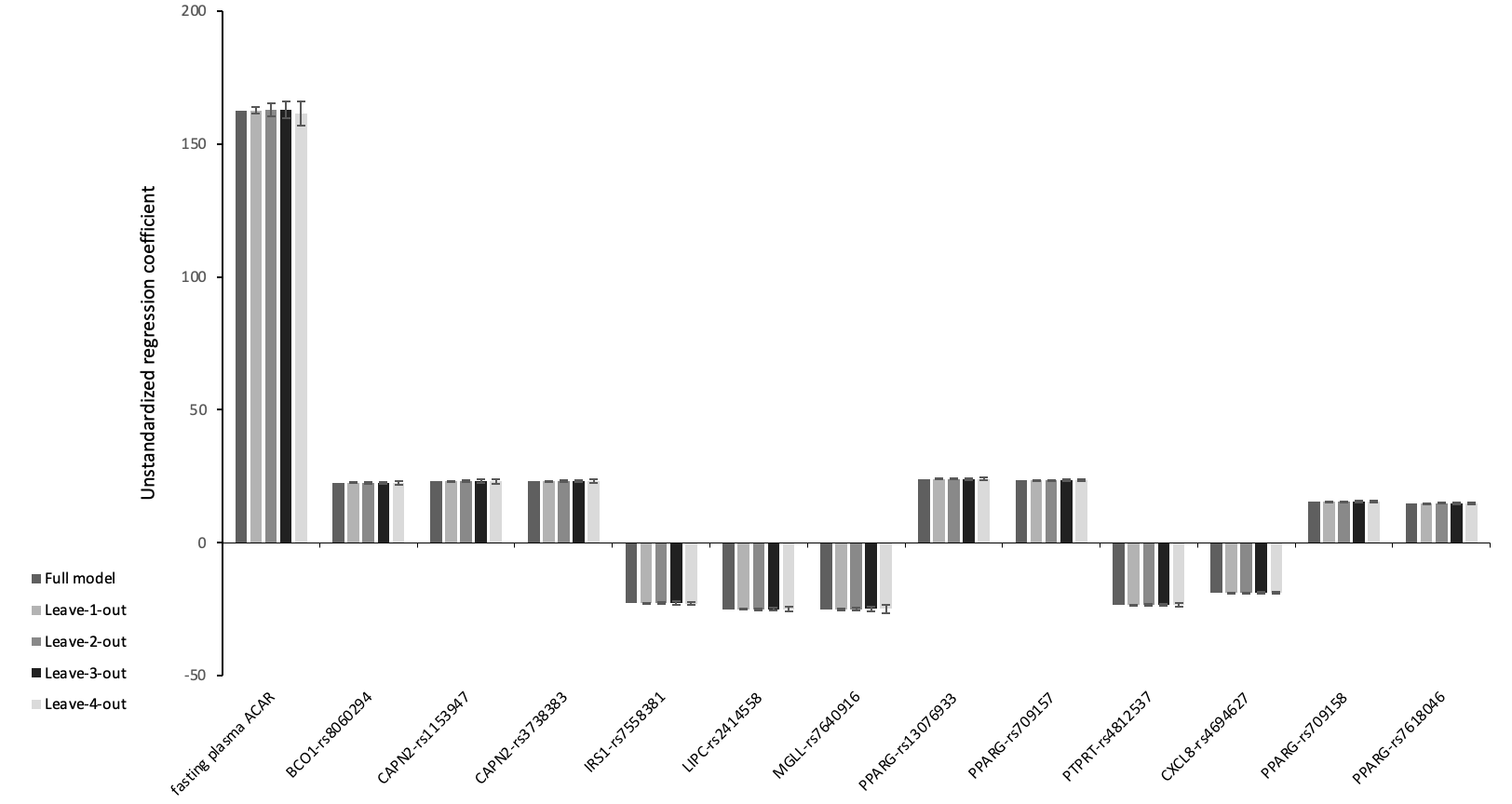


1. **ACAR**

**C. BCRY**


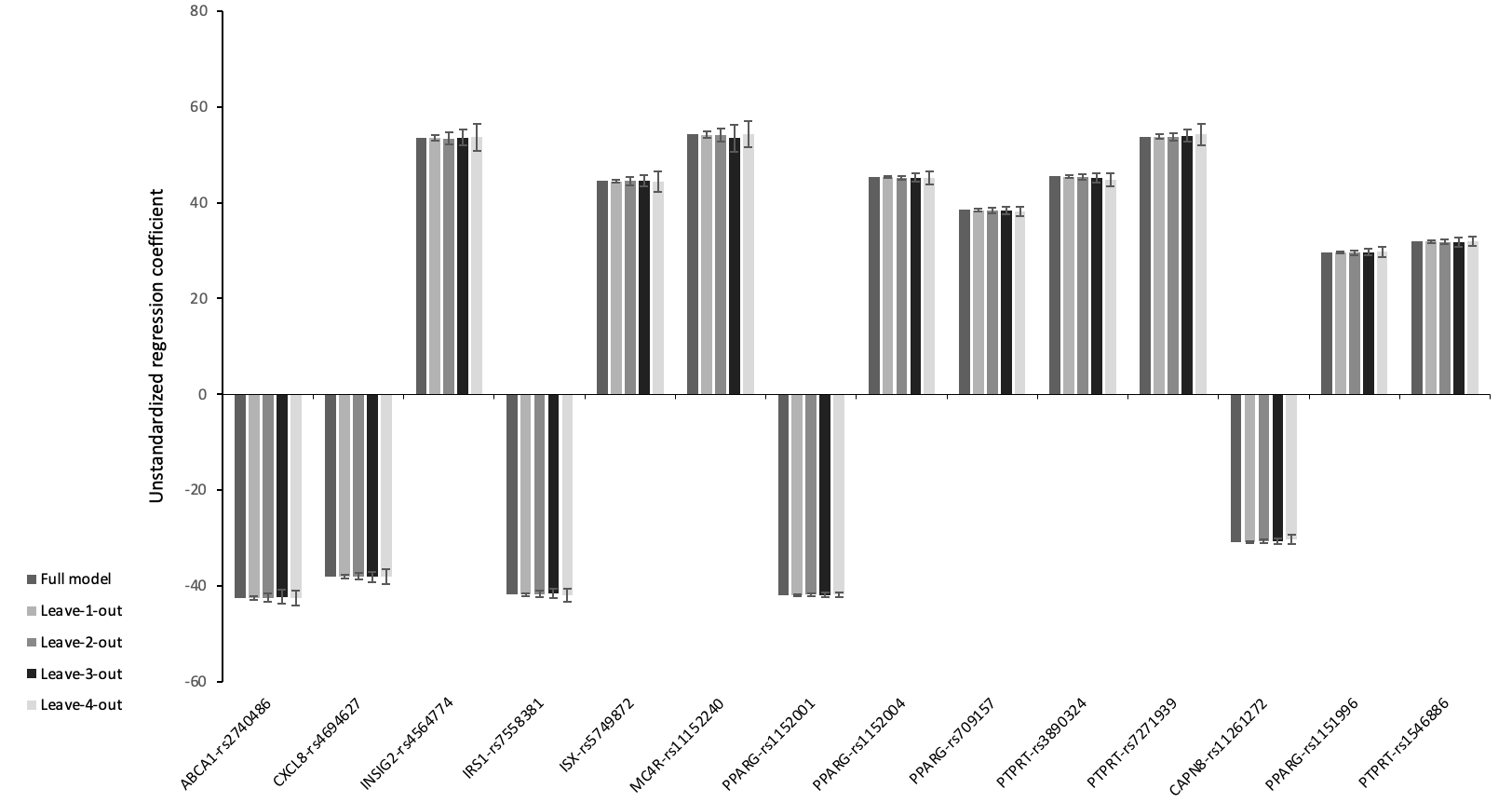


**D. Combined (BCAR, ACAR and BCRY) – Multi Y PLS regression model**

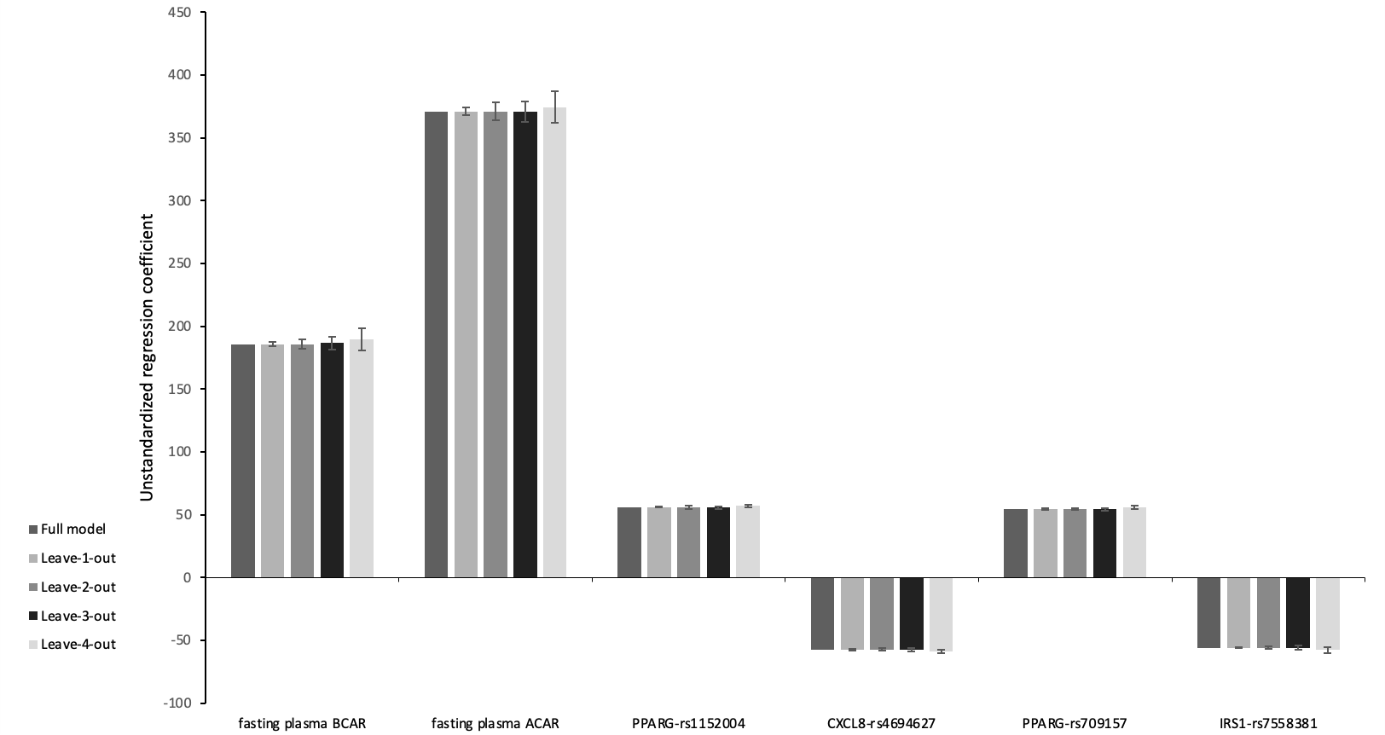
 **BCAR**

**ACAR**


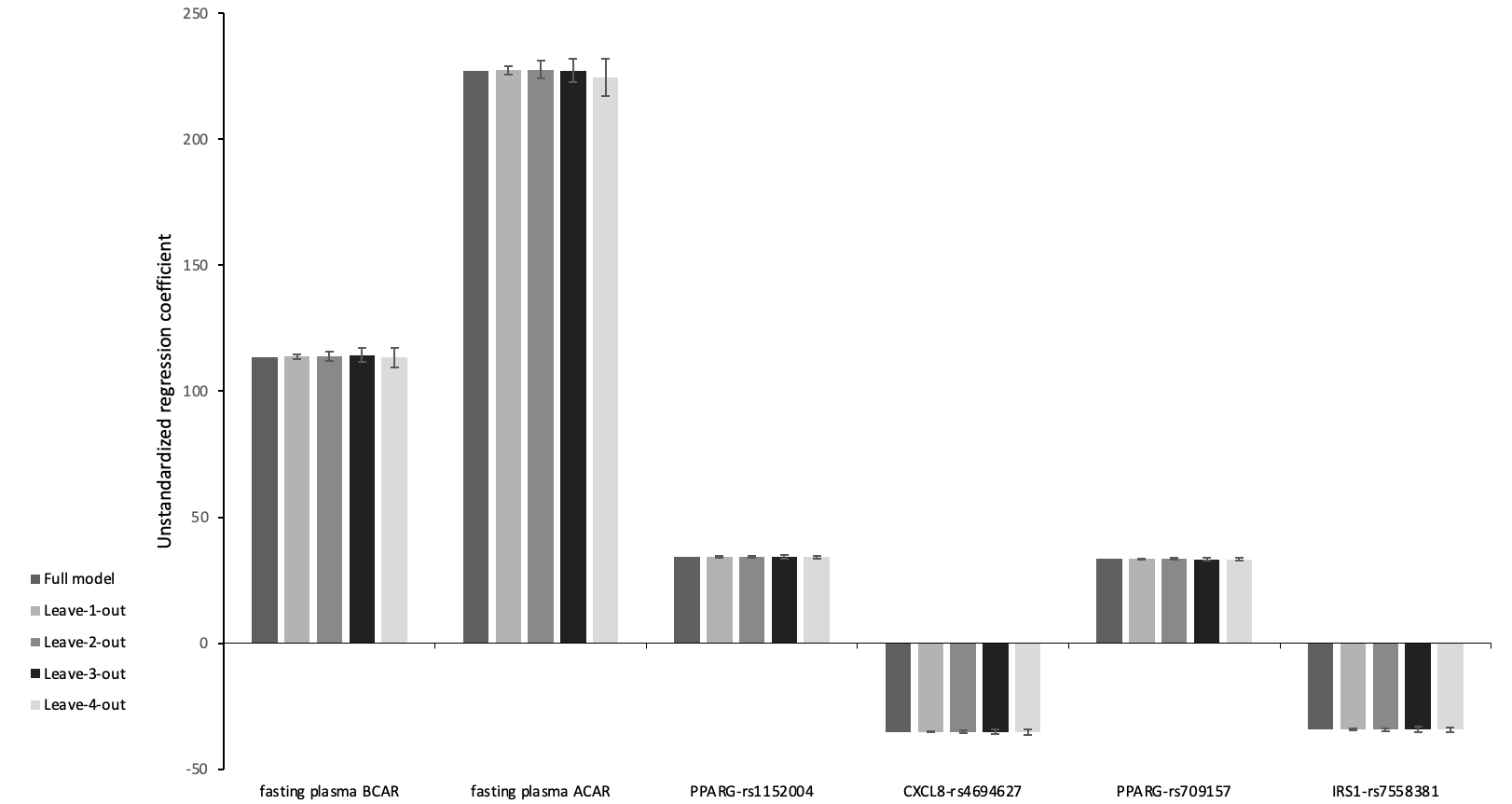


**BCRY**


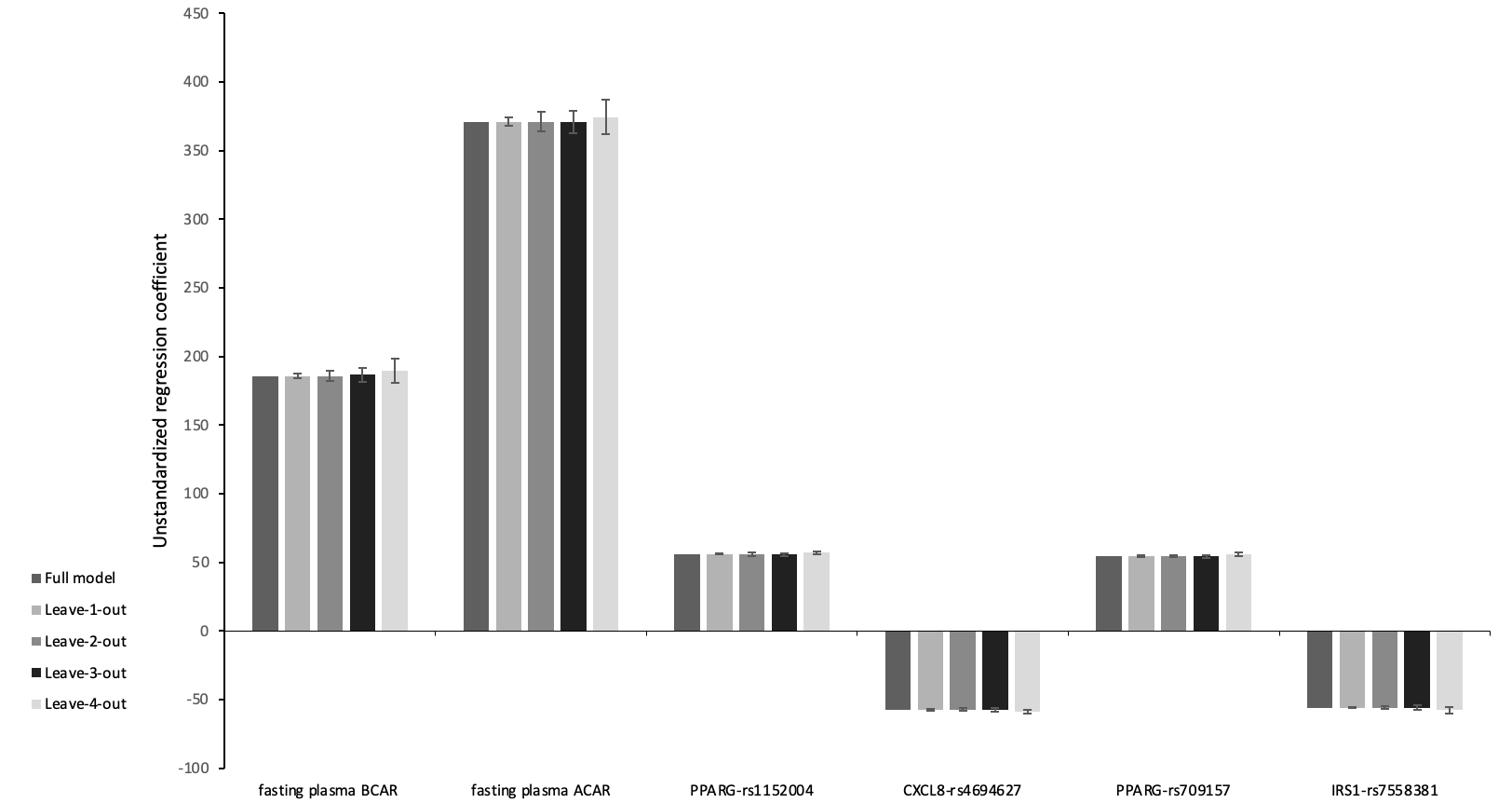


**Supplemental Figures S2:** SNPs stability following the leave-*k*-out procedure. *k* participants (*k*={1,2,3,4}) were randomly removed from the original dataset, thus leaving a training subset. These participants were then reintroduced in the training subset to assess the regression coefficients of the selected model. This test was performed as many times so that each participant was taken out once. One-way ANOVA performed for each gene showed no significant differences between the full model and the four training subsets generated by the procedure. Gene names are found in **Supplemental Table 1**. Abbreviations: BCAR – β-carotene ; ACAR – α-carotene; BCRY – β-cryptoxanthin.

1. R*^2^ and adjusted* R*² of the selected models after 100 permutations.*

This procedure 1) assesses the risk that the PLS regression model is spurious, *i.e.* the model fits the current data set well but does not predict *Y* well for new observations, and 2) tests for over-fitting. For over-fitting, the accuracy of fit (*R*^2^ and *R*² after cross-validation) of the original model was compared with the accuracy of fit of 100 models based on data where the order of the *Y* matrix for the participants (adipose tissue proVA CAR concentration) was randomly permuted, while the *X* matrix (the genotypes at the selected SNPs) was kept intact. Thus, a robust model (where the fit between *X* and *Y* is high) should be unable to predict the permuted *Y* variables with the intact *X* variables. **Supplemental Figure S3** shows the results of these permutations for the selected PLS regression models.


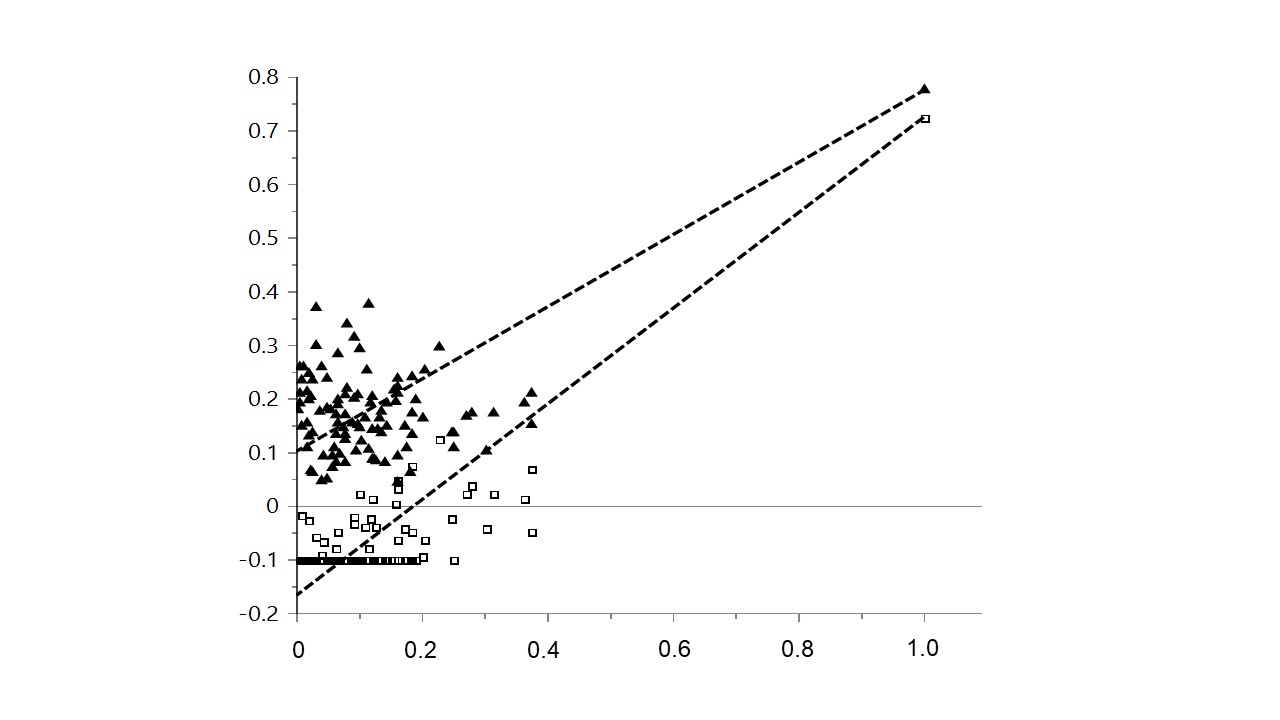


Original model

Permuted models

1. **BCAR**


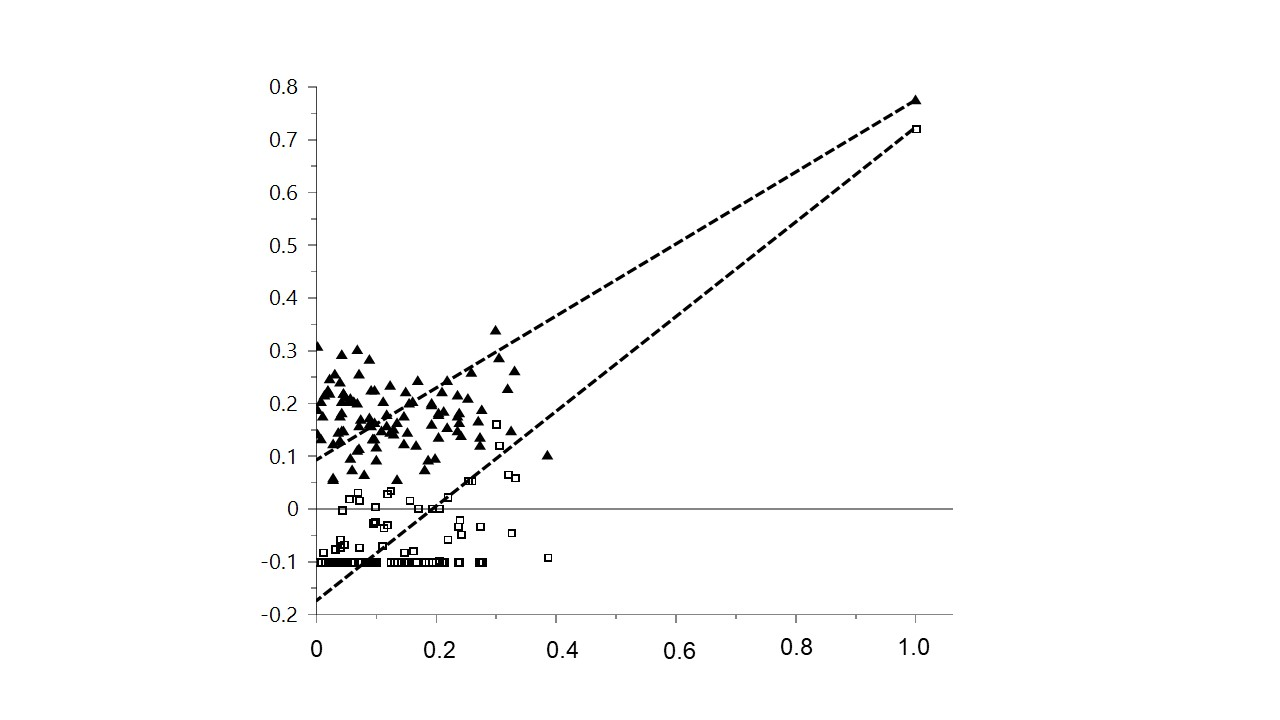


Original model

Permuted models

1. **ACAR**


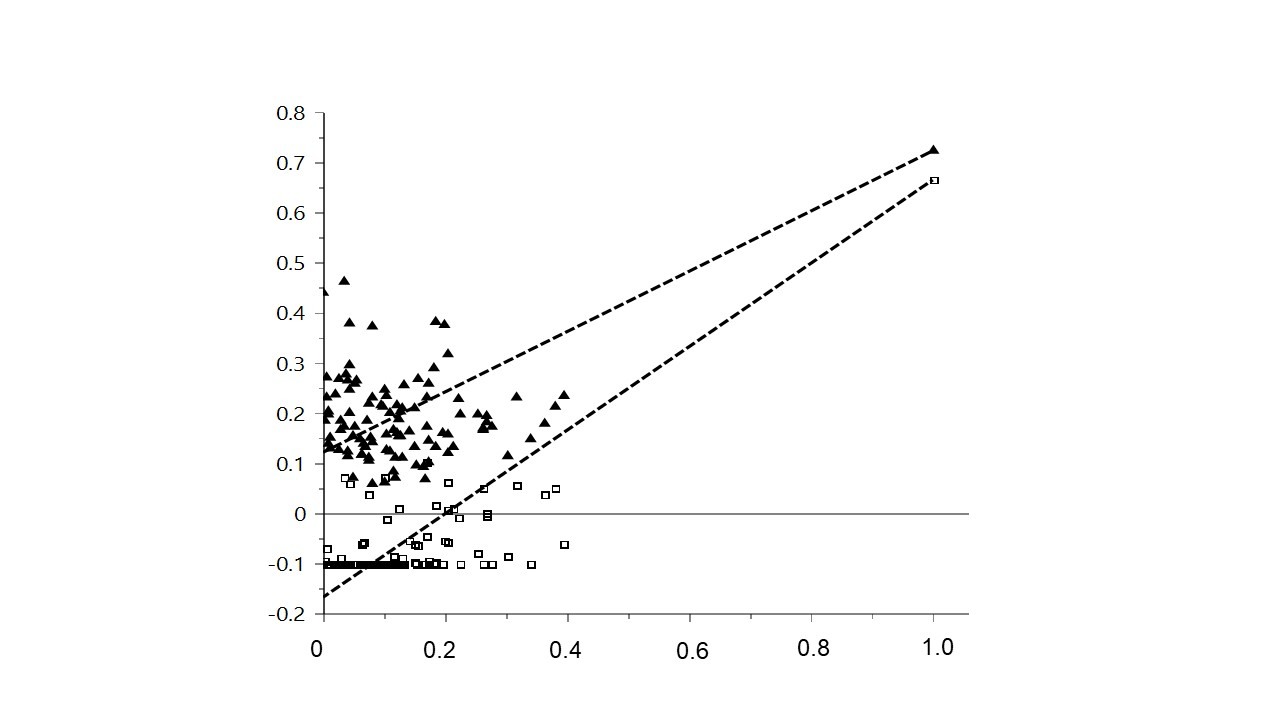


Original model

Permuted models

**C. BCRY**

Permuted models

**
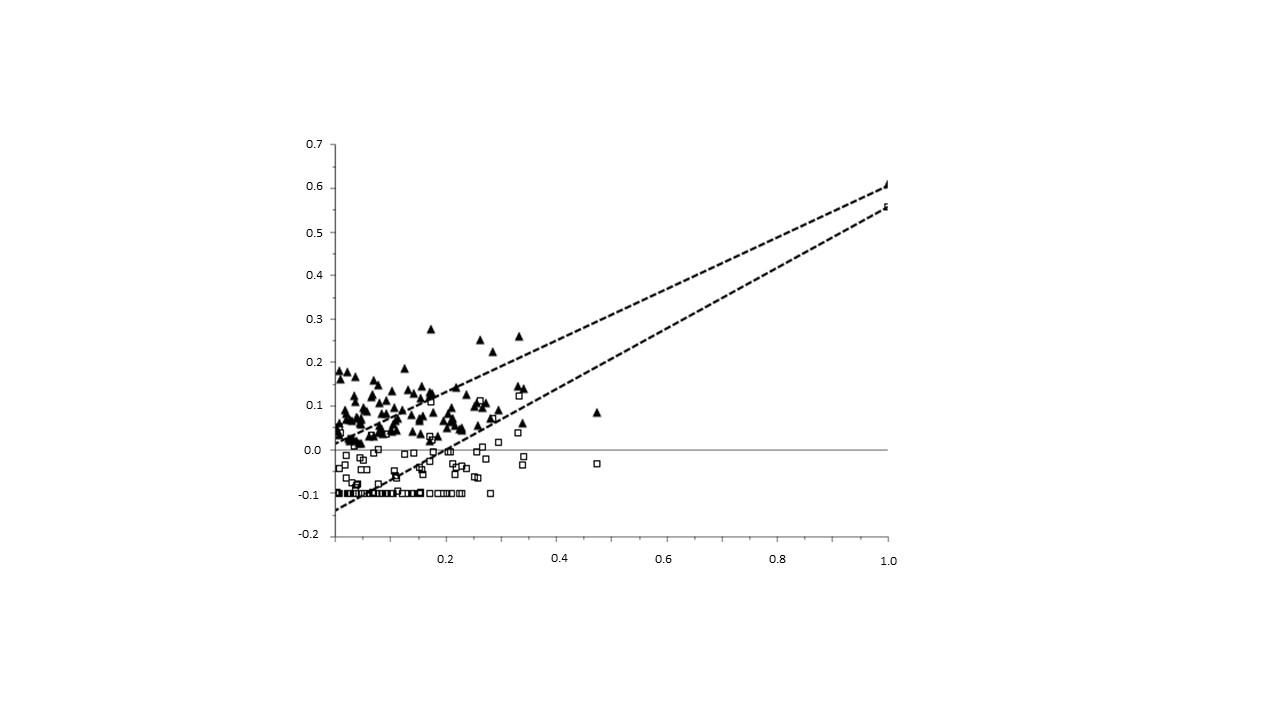
**

Original model

Permuted models

**D. Combined (BCAR, ACAR and BCRY) – Multi Y PLS regression model^a^**

**Combined (BCAR, ACAR and BCRY) – Multi Y PLS Model^a^**

**Supplemental Figure S3.** The horizontal axis represents the correlation between the permuted *Y*’s and the original *Y*’s. The vertical axis represents the *R*^2^ (dashed line and black triangles) and *R*² after cross-validation (dashed line and squares) values obtained in the permuted models. Values of the original model are on the far right (at correlation = 1), values of the 100 *Y*-permuted models are further to the left. This strongly supports the conclusion that the ability of the original, non-permuted model, to predict the adipose tissue proVA CAR concentration is not due to chance

**References:**

[1] W. de Haan, A. Bhattacharjee, P. Ruddle, M.H. Kang, M.R. Hayden, ABCA1 in adipocytes regulates adipose tissue lipid content, glucose tolerance, and insulin sensitivity, J Lipid Res 55(3) (2014) 516-23.

[2] J. Strychalski, A. Gugolek, E. Kaczorek-Lukowska, Z. Antoszkiewicz, P. Matusevicius, The BCO2 Genotype and the Expression of BCO1, BCO2, LRAT, and TTPA Genes in the Adipose Tissue and Brain of Rabbits Fed a Diet with Marigold Flower Extract, Int J Mol Sci 24(3) (2023) 2304.

[3] J. Amengual, G.P. Lobo, M. Golczak, H.N. Li, T. Klimova, C.L. Hoppel, A. Wyss, K. Palczewski, J. von Lintig, A mitochondrial enzyme degrades carotenoids and protects against oxidative stress, FASEB J 25(3) (2011) 948-59.

[4] C.C. Allred, T. Krennmayr, C. Koutsari, L. Zhou, A.H. Ali, M.D. Jensen, A novel ELISA for measuring CD36 protein in human adipose tissue, J Lipid Res 52(2) (2011) 408-15.

[5] H. Guillou, D. Zadravec, P.G. Martin, A. Jacobsson, The key roles of elongases and desaturases in mammalian fatty acid metabolism: Insights from transgenic mice, Prog Lipid Res 49(2) (2010) 186-99.

[6] S. Bandara, J. Moon, S. Ramkumar, J. von Lintig, ASTER-B regulates mitochondrial carotenoid transport and homeostasis, J Lipid Res 64(5) (2023) 100369.

[7] S. Bandara, S. Ramkumar, S. Imanishi, L.D. Thomas, O.B. Sawant, Y. Imanishi, J. von Lintig, Aster proteins mediate carotenoid transport in mammalian cells, Proc Natl Acad Sci U S A 119(15) (2022) e2200068119.

[8] M.S.F. Lavrador, M.S. Afonso, D.E. Cintra, M. Koike, V.S. Nunes, M. Demasi, C.J. Lin, L.M.M. Beda, L.A. Gioielli, R.P.A. Bombo, R.M. Machado, S. Catanozi, E.R. Nakandakare, A.M. Lottenberg, Interesterified Fats Induce Deleterious Effects on Adipose Tissue and Liver in LDLr-KO Mice, Nutrients 11(2) (2019) 466.

[9] X. Zhang, C.C. Zhang, H. Yang, K.G. Soni, S.P. Wang, G.A. Mitchell, J.W. Wu, An Epistatic Interaction between Pnpla2 and Lipe Reveals New Pathways of Adipose Tissue Lipolysis, Cells 8(5) (2019) 395.

[10] W.S. Blaner, J.C. Obunike, S.B. Kurlandsky, M. al-Haideri, R. Piantedosi, R.J. Deckelbaum, I.J. Goldberg, Lipoprotein lipase hydrolysis of retinyl ester. Possible implications for retinoid uptake by cells, J Biol Chem 269(24) (1994) 16559-65.

[11] M. Vaughan, J.E. Berger, D. Steinberg, Hormone-Sensitive Lipase and Monoglyceride Lipase Activities in Adipose Tissue, J Biol Chem 239(2) (1964) 401-9.

[12] P. Borel, C. Desmarchelier, M. Nowicki, R. Bott, Lycopene bioavailability is associated with a combination of genetic variants, Free Radic Biol Med 83 (2015) 238-44.

[13] A. Koppen, E. Kalkhoven, Brown vs white adipocytes: the PPARgamma coregulator story, FEBS Lett 584(15) (2010) 3250-9.

[14] L. Yvan-Charvet, A. Bobard, P. Bossard, F. Massiera, X. Rousset, G. Ailhaud, M. Teboul, P. Ferre, G. Dagher, A. Quignard-Boulange, In vivo evidence for a role of adipose tissue SR-BI in the nutritional and hormonal regulation of adiposity and cholesterol homeostasis, Arterioscler Thromb Vasc Biol 27(6) (2007) 1340-5.

[15] C.R. D'Adamo, V.J. Dawson, K.A. Ryan, L.M. Yerges-Armstrong, R.D. Semba, N.I. Steinle, B.D. Mitchell, A.R. Shuldiner, P.F. McArdle, The CAPN2/CAPN8 Locus on Chromosome 1q Is Associated with Variation in Serum Alpha-Carotene Concentrations, J Nutrigenet Nutrigenomics 9(5-6) (2016) 254-264.

[16] L. Ferrucci, J.R. Perry, A. Matteini, M. Perola, T. Tanaka, K. Silander, N. Rice, D. Melzer, A. Murray, C. Cluett, L.P. Fried, D. Albanes, A.M. Corsi, A. Cherubini, J. Guralnik, S. Bandinelli, A. Singleton, J. Virtamo, J. Walston, R.D. Semba, T.M. Frayling, Common variation in the beta-carotene 15,15'-monooxygenase 1 gene affects circulating levels of carotenoids: a genome-wide association study, Am J Hum Genet 84(2) (2009) 123-33.

[17] P. Borel, C. Desmarchelier, M. Nowicki, R. Bott, S. Morange, N. Lesavre, Interindividual variability of lutein bioavailability in healthy men: characterization, genetic variants involved, and relation with fasting plasma lutein concentration, Am J Clin Nutr 100(1) (2014) 168-75.

[18] L. Eriksson, J. Trygg, S. Wold, CV‐ANOVA for significance testing of PLS and OPLS® models, J Chemom 22(11-12) (2008) 594-600.

[19] E.W. Steyerberg, F.E. Harrell, Jr., G.J. Borsboom, M.J. Eijkemans, Y. Vergouwe, J.D. Habbema, Internal validation of predictive models: efficiency of some procedures for logistic regression analysis, J Clin Epidemiol 54(8) (2001) 774-81.
